# Supplementary material for: Azithromycin to prevent post-discharge morbidity and mortality in Kenyan children: a protocol for a randomised, double-blind, placebo-controlled trial (the Toto Bora trial)
Source: BMJ Open. 2017 Dec 29;7(12):e019170. doi: 10.1136/bmjopen-2017-019170 (PMC5778294; doi:10.1136/bmjopen-2017-019170)
Supplement: Author's manuscript [file bmjopen-2017-019170.draft_revisions.pdf]

BMJ Open is committed to open peer review. As part of this commitment we make the peer review history of every article we publish publicly available.

When an article is published we post the peer reviewers' comments and the authors' responses online. We also post the versions of the paper that were used during peer review. These are the versions that the peer review comments apply to.

The versions of the paper that follow are the versions that were submitted during the peer review process. They are not the versions of record or the final published versions. They should not be cited or distributed as the published version of this manuscript.

BMJ Open is an open access journal and the full, final, typeset and author-corrected version of record of the manuscript is available on our site with no access controls, subscription charges or pay-per-view fees (http://bmjopen.bmj.com).

If you have any questions on BMJ Open's open peer review process please email editorial.bmjopen@bmj.com

BMJ Open

Azithromycin to prevent post-discharge morbidity and mortality in Kenyan children: A protocol for a randomized, double-blind, placebo-controlled trial

Journal:	*BMJ Open*
Manuscript ID	bmjopen-2017-019170
Article Type:	Protocol
Date Submitted by the Author:	17-Aug-2017
Complete List of Authors:	Pavlinac, Patricia; University of Washington, Global Health<br>Singa, Benson; Kenya Medical Research Institute; Childhood Acute Illness and Nutrition Network<br>John-Stewart, G; University of Washington, Global Health, Epidemiology, Pediatrics, Allergy and Infectious Disease<br>Richardson, BA; University of Washington, Global Health, BIostatistics<br>Brander, Rebecca; University of Washington, Epidemiology<br>McGrath, Christine; University of Washington, Global Health<br>Tickell, Kirkby; University of Washington, Global Health; Childhood Acute Illness and Nutrition Network<br>Amondi, Mary; Kenya Medical Research Institute<br>Rwigi, Doreen; Kenya Medical Research Institute<br>Babigumira, Joseph; University of Washington, Global Health, Pharmacy<br>Kariuki, Sam; Kenya Medical Research Institute<br>Nduati, Ruth; University of Nairobi<br>Walson, Judd; University of Washington, Department of Global Health
**Primary Subject Heading**:	Global health
Secondary Subject Heading:	Paediatrics, Infectious diseases
Keywords:	Child mortality, Antibiotic prophylaxis, Post-discharge interventions

SCHOLARONE™
Manuscripts

Title: Azithromycin to prevent post-discharge morbidity and mortality in Kenyan children: A protocol for a randomized, double-blind, placebo-controlled trial

Running head: AZM to prevent post-discharge morbidity and mortality

Authors: Pavlinac PB[1], Singa BO[7,8], John-Stewart GC[1-4], Richardson BA[1,5], Brander RL[2], McGrath CJ[1], Tickell KD[1,8], Amondi M[7], Rwigi D[7], Babigumira JB[1,6], Kariuki S[7], Nduati R[9], Walson JL[1-4,8]

Affiliations: Departments of [1]Global Health, [2]Epidemiology, [3]Pediatrics, [4]Allergy and Infectious Disease, [5]Biostatistics, [6]Pharmacy, University of Washington, Seattle, WA, USA; [7]Kenya Medical Research Institute, Nairobi, Kenya; [8]The Childhood Acute Illness & Nutrition Network, Nairobi, Kenya; [9] University of Nairobi, Nairobi, Kenya

Correspondence: Patricia Pavlinac, 325 9th Ave, Box 359931, Seattle, WA 98104 (ppav@uw.edu)

Sponsored by: This trial is sponsored by the National Institutes of Health Eunice Kennedy Shriver National Institute of Child Health & Human Development (R01 HD079695) and drug donation by Pfizer IIR #WI201906.

Key words: child mortality, antibiotic prophylaxis, post-discharge interventions

Word count: 5872

For peer review only

ABSTRACT

Introduction: Child mortality due to infectious diseases remains unacceptably high in much of sub-Saharan Africa. Children who are hospitalized represent a particularly vulnerable population, both during and following hospitalization. Children being discharged from hospital represent an accessible high-risk population in which targeted use of antibiotics could offer clinical benefit.

Methods and analysis: In this randomized, double-blind, placebo-controlled trial, 1400 children aged 1 to 59 months discharged from 2 hospitals in western Kenya, in Kisii and Homa Bay, will be randomized to either a 5-day course of azithromycin or placebo to determine whether a short-course of azithromycin reduces rates of re-hospitalization and/or death in the subsequent 6-month period. The primary analysis will be modified intention-to-treat and will compare the rates of re-hospitalization or death in children treated with azithromycin or placebo using Cox proportional hazard regression. The trial will also explore mechanistic questions including the effect of a short course of azithromycin on enteric and nasopharyngeal infections and cause-specific re-hospitalizations. We will also identify clinical and host risk determinants of post-discharge morbidity and mortality. The emergence of antibiotic resistance among treated individuals and in a random subset of their primary caregivers will also be assessed and cost-effectiveness analyses performed to inform policy decisions.

Ethics and dissemination: Study procedures were reviewed and approved by the institutional review boards of the Kenya Medical Research Institute, the University of Washington, and the Kenyan Pharmacy and Poisons Board. The study is being externally monitored by Westat® and a data safety and monitoring committee has been assembled to monitor patient safety and evaluate the efficacy of the intervention. The results of this trial will be published in peer-reviewed scientific journals and presented at relevant academic conferences and to key stakeholders.

Trial registration number: NCT02414399

Key words: child mortality, antibiotic prophylaxis, post-discharge interventions

STRENGTHS AND LIMITATIONS OF THIS STUDY

- Randomized, placebo-controlled, double-blinded design and intention-to-treat analysis plan will ensure unbiased treatment effect measure
- Comprehensive data are collected, including biological specimens for all child participants and a subset of adult caregivers, for analyses of mechanisms of post-discharge morbidity and mortality, as well as assessments of antibiotic resistance and cost-effectiveness
- Results will likely be generalizable due to the limited exclusion criteria, large sample size, and multiple study sites
- Causes of death and re-hospitalization may not be accurate due to limited availability of medical records and recall bias in caregiver report
- The primary endpoint of this study is a combined outcome of re-hospitalization and death, which, while improving statistical power, may present challenges for interpretation

For peer review only

BACKGROUND

An estimated 3.5 million deaths occur annually in children less than 5 years of age in sub-Saharan Africa (SSA), approximately 70% of which are due to infectious causes.[1] One-year mortality rates as high as 15% have been documented following hospital discharge in SSA, a rate that is 8-fold higher than similarly-aged children in the community.[2-4] Children being discharged from hospital in SSA may represent an accessible high-risk population in which to target interventions to reduce mortality.

A single dose of azithromycin halved mortality rates in among Ethiopian children living in communities randomized to receive the antibiotic as part of a mass drug administration program.[5, 6] However, concerns about the potential for the emergence of antimicrobial resistance, possible toxicity, and feasibility of delivery are barriers to community-wide distribution of antibiotics. Targeted antimicrobial interventions, including the use of cotrimoxazole among HIV-infected children and the use of amoxicillin or cefdinir among malnourished children, have been shown to reduce mortality in these specific vulnerable populations.[7-10] Children who have been recently hospitalized are a high-risk population in which targeted azithromycin distribution may optimize benefit while reducing both individual and population level risks.

Among high-risk pediatric populations with history of recent illness, azithromycin may treat residual disease not eliminated during inpatient therapy, may provide prophylaxis against infectious exposures during a time of immune vulnerability following illness, and may reduce carriage of pathogenic organisms, including those associated with mucosal surface disruption, inflammation, and immune activation.

OBJECTIVE

The primary objective of this double-blind, placebo-controlled, randomized controlled trial (RCT) is to determine whether a 5-day course of azithromycin in children age 1 to 59 months discharged from hospital in western Kenya reduces rates of re-hospitalizations and/or death in the subsequent six months. The secondary objectives are (1) to evaluate possible mechanism(s) by which azithromycin may affect morbidity and mortality, by comparing reasons for re-hospitalization and prevalence of enteric and nasopharyngeal infections between the randomization arms; (2) to determine whether empiric administration of azithromycin at hospital discharge increases risk of antimicrobial resistance in commensal *Escherichia coli* (*E. coli*) and *Streptococcus pneumoniae (S. pneumoniae)* isolates from treated children and their primary caregiver; (3) to identify correlates and intermediate markers of post-discharge mortality and hospital readmission; (4) to determine the cost-effectiveness of post-discharge administration of a 5-day course of azithromycin in settings of varying antibiotic use, re-hospitalization rates, and mortality rates; and (5) to create a repository of stool, nasopharyngeal, and blood specimens from highly characterized, recently discharged children to be used to address future research questions.

METHODS

Reporting of this study protocol has been verified in accordance with the SPIRIT (Standard Protocol Items for Randomized Trials) recommendations.

Eligibility

Children age 1 to 59 months old weighing at least 2 kg and have been hospitalized and subsequently discharged and who are willing to participate will be eligible for inclusion. Children will be excluded if: azithromycin is contraindicated (children taking or prescribed other macrolide antibiotics, such as erythromycin or clarithromycin, or the protease inhibitor, lopinavir); they were admitted to hospital for a trauma, injury, or a birth defect; they do not plan to remain in the study site catchment area for at least 6 months; the legal guardian does not provide consent; or if a sibling was enrolled in the Toto Bora Trial on the same day of discharge. Caregivers of potentially eligible children must be at least 18 years of age or classified as an emancipated minor and be willing to participate in the Adult Contact Cohort if randomly selected.

Recruitment

Children will be recruited from the inpatient wards of Kisii Teaching & Referral and Homa Bay County Hospitals where study staff will accompany hospital staff on ward rounds to identify children being discharged each day. All discharged children, as determined by the onsite hospital clinicians, will be screened by study staff during working hours. If the caregiver is interested in participating and indicates consent for screening, the study staff will screen the child for eligibility, and if eligible, will obtain informed consent for study participation.

For peer review only

Informed consent includes an explanation of the potential risks and benefits of the study and additional provision for use of participant data and samples for future studies, and will be conducted in the language of the respondent's choosing (English, Kiswahili, Kisii, or Luo). The parent or guardian (primary caregiver) must sign written informed consent (or provide a witnessed thumbprint if not literate) prior to enrollment.

Enrollment

Children will be enrolled at the time of discharge by the clinical staff. At enrollment, primary caregivers will be interviewed to assess demographic information, medical history, and detailed contact information for the child. Medical records will also be used to abstract information from the hospitalization (including presenting diagnosis, medical management, length of stay, procedures performed, relevant medical history, physical examination, and laboratory data). All enrolled participants will undergo a physical examination performed by the study clinician, including measurement of height/length, weight, and mid-upper arm circumference (MUAC), each of which will be measured three times. The height, weight, and MUAC of the caregiver will also be collected. HIV status will be obtained from medical records or from performed testing if records are not available. Detailed home location and contact information will be collected to enable patient tracing.

Specimen collection

Specimens will be collected at enrollment (prior to study medication administration, as well as at 3 and 6-month follow-up visits). All children will also be asked to provide a whole stool for enteric pathogen identification and storage. Stool samples/swabs will be divided within one hour of collection for the following purposes: 1) placed in Cary-Blair for eventual bacterial culture (FecalSwab Cary-Blair Collection and Transport Systemt™, Copan Diagnostics), 2) immediately tested for *Giardia* and *Cryptosporidium* using the immunoassay (Quik Chek™, Alere) and 3) placed in -80°C storage for future molecular determination of pathogen or commensal flora and markers of gut function (whole stool for these purposes will be divided into two separate vials). If a child cannot produce whole stool, two flocked rectal swabs (Pedatric FLOQswab™, Copan Diagnostics) will be collected and one placed in Cary-Blair and the other stored in -80°C for future analyses.

One flocked swab (Nylon Flocked Dry Swabs, Copan Diagnostics) nasopharyngeal swab will also be collected from all enrolled children at each time point, immediately placed in skim milk, tryptone, glucose, and glycerine (STGG) media, and frozen (-80°C) within 1 hour of collection for future *S. pneumoniae* culture. [11, 12] Primary caregivers in the Contact Cohort will also be asked to provide a stool sample (or 2 rectal swabs) and nasopharyngeal sample at each visit for testing and storage as described above.

Venous blood (up to 1 teaspoon [5mL]) will be collected from all enrolled children and caregivers enrolled in the Contact Cohort at each at each time point into EDTA tube and separated for the following purposes: 1) 0.5mL for immediate HIV-testing (if indicated according to Kenyan Ministry of Health guidelines), 2) 0.4mL for a thin malaria smear which will be stored at room temperature, 3) 0.4mL for a dried blood spot whole blood -80°C storage and eventual sickle cell testing and 4) 2-4mL for plasma and buffy coat isolation and -80°C storage.

Randomization

Block randomization (1:1) in random sized blocks of no more than 10, stratified by site, will be used. Primary randomization will include allocation to the Contact Cohorts at a ratio of 1:5 (resulting in 150 per treatment arm). Each subject will be assigned a Patient Identification Number (PID), and the randomization code linking each PID to the allocated treatment will be maintained by the University of Washington Research Pharmacy. Study participants, investigators, the study staff, hospital clinicians, and persons involved in data management or analysis will remain blinded to the allocation group during all data collection phases of the study.

Intervention

Caregivers of enrolled children will be provided a 5-day course of oral suspension formulation azithromycin (Zithromax® from Pfizer, 10 mg/kg on day 1, followed by 5mg/kg/day on days 2-5) or identically appearing and tasting placebo at discharge. Dosing ranges were determined such that a given child would never be under-dosed and not over-dosed by more than 20% that the weight-specific intended dose (Table 1). The day 1 dose will be split in half and the first half administered first by study clinician (to be observed by the

For peer review only

caregiver) followed by the second half administered by the caregiver under careful observation of the study staff. Caregivers will be provided with visual instructions in the language of their choosing (English, Kiswahili, Luo, Kisii).

Automated daily text message reminders will be sent for the four days following discharge and caregivers asked to respond with whether or not the child took the daily dose. The response text message will be free of charge to caregivers and caregivers will be reimbursed for each response at the final study visit. Caregivers are also asked to record each administered dose on the bottle and to return bottles at the 3 month follow-up visit.

Table 1. Azithromycin dosing chart by child weight

Weight (kg)	Day 1 dose (mL)	Day 2-5 dose (mL)
**2.0**	0.25 x 2	0.25
**2.1-2.4**	0.30 x 2	0.30
**2.5-2.8**	0.35 x 2	0.35
**2.9-3.2**	0.40 x 2	0.40
**3.3-3.6**	0.45 x 2	0.45
**3.7-4.0**	0.50 x 2	0.50
**4.1-4.8**	0.60 x 2	0.6
**4.9-5.6**	0.70 x 2	0.7
**5.7-6.8**	0.85 x 2	0.85
**6.9-8.0**	1.0 x 2	1.0
**8.1-9.6**	1.2 x 2	1.2
**9.7-11.2**	1.4 x 2	1.4
**11.3-13.6**	1.6 x 2	1.6
**13.7-16.0**	2.0 x 2	2.0
**16.1-19.2**	2.4 x 2	2.4
**19.3-23.2**	2.9 x 2	2.9
**23.3-25.0**	3.2 x 2	3.2

Follow-up Procedures

All enrolled children and primary caregivers will be scheduled to return to the health facility at 3 and 6 months following enrollment to collect clinical information and samples. Anthropometric measurements will be obtained from all children and caregivers at both follow up visits (height/length, weight, MUAC) and caregivers will be asked about any hospitalizations occurring since the last time the child was seen by study staff. A flowchart of follow-up and sample collection is shown in Figure 5. Caregivers will be provided with 400KSH (approximately $4USD) to cover the cost of their round-trip transportation.

Transportation cost will be reimbursed at each follow-up visit. If the participant does not return at their scheduled time, study staff will attempt to make contact with the primary caregiver via cell phone; if no telephone number is provided, or if the participant cannot be reached, study staff will trace the child to the household within 2 weeks of the scheduled follow-up time.

During scheduled follow-up visits, study staff will use a standardized questionnaire to ascertain history of recent illness/morbidity, post-discharge medication use including antibiotic treatment, and current condition of the child (any hospitalizations, admission and discharge date of any hospitalization, alive or dead, date of death if applicable). If caregivers report a hospitalization, causes of admission, medication administration, and length of stay will be ascertained from both caregivers and medical records, when available.

Caregivers will be encouraged, at enrollment and at each subsequent contact, to bring the child to the study health facility at any time the child is sick. Study staff will triage children to the appropriate health facility staff and will conduct a brief unscheduled visit questionnaire to ascertain adverse event information. If the unscheduled visit leads to a hospitalization, this will trigger the completion of a hospital admission form.

If at any point during follow-up a child dies, a verbal autopsy using the shortened Population Health Metrics Research Consortium questionnaire will be performed[13] If the death occurred in a hospital, data from the hospital records, including cause of death, if available, will be abstracted. If a death certificate is available, cause(s) and timing of death will be abstracted.

Final causes of re-hospitalization and death will be determined after data collection is complete by an independent adjudication committee comprised of clinicians specializing in pediatrics and infectious disease. Sources of cause of re-hospitalization (medical records and caregiver report) and causes of death (causes automatically assigned from the verbal autopsy using SmartVA-Analyze [Tariff 2.0 Method][14], hospital records, or death certificates) will be presented to the adjudication committee for final cause assignment.

Laboratory procedures and specimen collection and storage

Stool (rectal swabs), nasopharyngeal swabs and blood will be collected as described above and undergo either immediate or future laboratory testing as described in Table 2. All biological samples will be collected by staff trained in biosafety and Good Clinical Laboratory Practice (GCLP). Samples will be processed in Kenya when technology is available at one of the following laboratories: Kenya Medical Research Institute (KEMRI) (Wellcome Trust or Centre for Microbiology Research [CMR]) or at the University of Nairobi (Microbiology Department). Metagenomic analyses and/or analyses that require technology not available in

For peer review only

Kenya will be performed at the University of Washington. If stool culture results report *Shigella* or Salmonella infection, the study staff will contact the child's caregiver and encourage the caregiver to bring the child back for an evaluation and treatment if the child is symptomatic.

Table 2. Sample processing description.

Specimen Collected	Purpose	Tests Performed
Stool/ flocked rectal swabs	Bacterial ID and storage for AST	Fresh samples/rectal swabs will be cultured to identify *Shigella*, *Salmonella*, *Campylobacter*, and *Escherichia coli* using standard microbiologic methods and biochemically confirmed using bioMérieux's API® strips. All *Shigella*, *Salmonella*, and *Campylobacter* isolates, as well as a random subset of *E.coli* isolates will undergo antimicrobial resistance testing using disc diffusion for the following antibiotics: amoxicillin-clavulanic acid (augmentin), ampicillin, azithromycin, chloramphenicol, ciprofloxacin, ceftriaxone, ceftazidime, cefoxitin, gentamicin, imipenem, trimethoprim-sulfamethoxazole, ceftazidime/clavulanate (ESBL), cefotaxime/clavulanate (ESBL). Categorizations of susceptible, intermediate, and resistant will be determined using zone-size cut-offs outlined in CLSI interpretive standards (M100-S24 2014).
	Parasite detection	Fresh stool and rectal swabs will be tested for *Giardia* and *Cryptosporidium* using the immunoassay Giardia/ Cryptosporidium QUIK CHEK™.
	Storage	Stool/ flocked swabs and colonies of *E.coli*, *Shigella* spp., *Salmonella* spp., and *Campylobacte*r spp. will be stored at -80°C.
Nasopharyngeal Swabs	Bacterial isolation, storage, and resistance testing	*Streptococcus pneumoniae* (*S. pneumoniae*) colonies will be isolated using standard microbiologic or molecular diagnostic protocols and susceptibility testing performed using standard microbiologic or molecular techniques. A random subset of *S. pneumoniae* isolates will undergo antimicrobial resistance testing using disc diffusion for the following antibiotics: amoxicillin-clavulanic acid (Augmentin), ampicillin, azithromycin, chloramphenicol, ciprofloxacin, ceftriaxone, ceftazidime, cefoxitin, gentamicin, imipenem, and trimethoprim-sulfamethoxazole. Categorizations of susceptible, intermediate, and resistant will be determined using zone-size cut-offs outlined in CLSI interpretive standards M100-S24 2014.
	Storage	Back-up sample and *S.pneumoniae* colonies will be will be stored at -80°C.
Blood	HIV and malaria testing	HIV testing will be performed per Kenyan National Guidelines and malaria microscopy performed using standard methods.
	Storage	Plasma and buffy coat will be stored at -80°C. Dried blood spots will be stored at room temperature.

Data Management and Confidentiality

Personal information about the participants, including medical records and data ascertained per caregiver interview, will be securely stored in files in the study offices at the study sites. Only pre-designated study staff will have access to the files. Data will be entered into an electronic database (Dacima® Electronic Data Capture) regularly by study staff. Access to the electronic database will be secured using password protected accounts for study staff. Data reports of screening, enrollment, and exclusion totals will be disseminated to the study team on a weekly basis; reports including baseline demographic characteristics, laboratory results, adherence data, and serious adverse event summaries will be distributed to study co-investigators and data monitors quarterly. Data will be regularly queried to facilitate ongoing data cleaning.

Data Analysis

Primary endpoints:

The primary study endpoint is a combined outcome of mortality and hospital readmission, as re-hospitalization is highly associated with risk of subsequent poor outcome. Re-hospitalizations that are a continuation of management from the previous hospitalization (such as elective blood transfusion) or that occur during enrollment procedures, due to a clinical deterioration post-discharge, will be excluded from the analysis. Loss to follow-up will be defined as non-attendance at both follow-up visits despite up to one month of active tracing and no clear evidence of death.

Secondary endpoints include:

1. <u>*Cause-specific re-hospitalizations*</u> assessed by questionnaire (maternal recall of diagnosis) at day 90 and day 180 follow-up visits and medical record review (discharge diagnosis). In cases when both sources are

For peer review only - http://bmjopen.bmj.com/site/about/guidelines.xhtml

available, information from the medical record will be considered superior. Separate analyses will be performed for each diagnosis: diarrhea, acute respiratory infection, malnutrition, or malaria.

2. <u>*Enteric pathogen carriage*</u>**,** operationalized as presence of a bacterial pathogen-*Shigella* species (spp.), *Campylobacter* spp., or *Salmonella* spp., or parasite- *Giardia* or *Cryptosporidium* in stool or rectal swabs assessed at day 90 and day 180 follow-up visits.
3. <u>*Streptococcus pneumoniae (S. pneumoniae)*</u> isolated from nasopharyngeal swab cultures at 90 and 180-day follow-up visits.
4. <u>*Antimicrobial resistance*</u>**,** specifically resistance to azithromycin, ampicillin, augmentin, trimethoprim-sulfamethoxazole, in *E.coli* and *S. pneumoniae* isolates, and presence of ESBL in *E.coli* isolates, from day 90 and day 180 samples.

<u>*To compare rates of re-hospitalization and mortality in the 6 months following hospital discharge among Kenyan children receiving 5-day azithromycin vs. placebo*</u>**.** Primary analyses will be modified intent-to-treat (mITT) based on randomization allocation to the 5-day course of azithromycin versus placebo. Cumulative incidence of death or first re-hospitalization will be compared between treatment groups using Cox proportional hazards regression. Participants will be censored at the date of their first re-hospitalization, or at the date of death. Median time to hospitalization-free survival will be compared between randomization groups using Kaplan-Meier (K-M) survival analysis and associated log-rank test. If the baseline assessment of randomization reveals an imbalance in characteristics between the treatment groups, we will evaluate these variables as potential confounders in a sub-analysis secondary to the mITT. Potential baseline confounders will be added stepwise in a multivariable Cox model and maintained in the model if adjustment changes the hazard ratio by more than 10%. In per protocol analyses also secondary to the mITT, we will compare treatment effects in groups defined by self-reported adherence to the 5-day course of azithromycin (5 doses vs. < 5 doses; ≥3 doses vs. <3 doses; >1 dose vs. 1 dose only). In addition, we will conduct Cox regression and K-M survival analyses for time to mortality and time to re-hospitalization as separate endpoints to understand intervention effects on these outcomes individually. The assumption of proportional hazards will be checked in all models using graphical methods including plotting a ln(-ln(S(t))) plot for each treatment group and assessing the parallelism of the two lines and by plotting Schoenfeld residuals over time. If there is substantial missing covariate data, multiple imputation using the Markov chain Monte Carlo (MCMC) method will be used to impute covariate information. Missing outcome data (death or re-hospitalization) will not be imputed, but participants will be censored at the last follow-up visit therefore contributing some person-time to the analysis.

<u>*To evaluate possible mechanism(s) by which azithromycin may affect morbidity and mortality, by comparing reasons for re-hospitalization and change in prevalence of pathogen carriage between the randomization arms.*</u> To evaluate the association between azithromycin and the rates of cause-specific re-hospitalizations (hospitalization due to diarrhea, acute respiratory infection, malnutrition, or malaria) we will use Anderson-Gill proportional hazards modeling with previous re-hospitalizations included as time-dependent covariates in the model to capture the dependent structure of recurrence times. Because we will not have granularity in the time points other than 90-days and 6-months for assessment of pathogen carriage, we will compare the prevalence of a bacterial and parasitic pathogens (*Shigella*, *Salmonella*, *Campylobacter, Cryptosporidium, Giardia*) at 90-days and 6 months by randomization arm using generalized estimating equations (GEE) with a Poisson link, exchangeable correlation structure, and will adjust for baseline presence of a bacterial pathogen. To determine whether an observed association between the intervention and pathogen carriage wanes over time, we will test the hypothesis that the prevalence ratios comparing carriage in intervention arms are the same at the two follow-up time points using a chi-squared test.

<u>*To determine whether empiric administration of azithromycin at hospital discharge increases risk of antimicrobial resistance in commensal E. coli and pneumococcal isolates from treated children and their household contacts.*</u> Among children and adult household contacts in whom commensal *E. coli* and/or *S. pneumoniae* are isolated, we will compare the proportion of isolates resistant to azithromycin, ampicillin, augmentin, and trimethoprim-sulfamethoxazole, between randomization arms and Contact Cohorts for each arm, at 90-days and 6 months using GEE with a Poisson link and exchangeable correlation structure. A chi-squared test will be used to determine whether the association between intervention arm and antimicrobial resistance wanes over time. Because the likelihood of having a bacterial pathogen isolated may depend on baseline factors, including intervention arm, we will conduct secondary analyses utilizing propensity scores to

account for the potential differential likelihood of having antimicrobial susceptibility testing performed, which will allow us to make inference to the entire study population and their contacts.

To identify correlates and intermediate markers of post-discharge mortality and hospital-readmission among hospitalized children. Enrollment hospital admission diagnosis, indicators of malnutrition, age, HIV-exposure and HIV-infection status, sickle cell anemia, and randomization arm will be assessed in a multivariable Cox regression model to identify correlates of the primary endpoint of death and/or hospital-readmission independent of the treatment effect. In addition, Cox regression models will also be built for correlates of mortality and correlates of re-admission individually to understand distinct cofactors for each of these outcomes.

To determine the cost-effectiveness of post-discharge administration of a 5-day course of azithromycin in settings of varying antibiotic use, re-hospitalization rates, and mortality rates. Costs analysis: We will assess the costs of all supplies, services and equipment necessary to implement the intervention (direct medical costs). The perspective will be that of the healthcare provider, i.e. Kenya's Ministry of Health. Using WHO guidelines and its ingredients approach, we will quantify the resources and associated unit costs required to deliver a 5-day course of azithromycin, organized in standard expenditure categories: personnel (salaries), supplies including drugs, equipment, services, space and overhead. We will also measure the costs of severe child hospitalizations, the costs for the different types of personnel employed (e.g. nurses/doctors) and the time demanded from them for conducting the intervention.[15] When data are missing, they will be complemented by data extracted from the literature and other available sources. Full incremental costs will be derived, with estimation of the potential healthcare cost-offset realized in avoiding severe hospitalizations. Costs will be measured in local currency (Kenyan Shilling) and converted into US$. Our main metric will be cost per child treated. Cost-effectiveness analysis (CEA): we will develop a CEA mathematical model, and estimate incremental costs and cost-effectiveness for implementation of the intervention. The model will include two components: costs (described immediately above) and health benefits. The study will provide clinical outcomes (mortality/morbidity) over a 6-month follow-up period. Subsequently, deaths averted, life-years saved and disability-adjusted life years (DALYs) averted by the intervention will be estimated. We will estimate: a) incremental costs and b) incremental cost-effectiveness of the intervention vs. status quo. *Incremental costs* are the net sum of the costs to implement the intervention compared with status quo, and the costs averted due to the decrease in severe child hospitalizations. *Incremental cost-effectiveness* ratios (ICERs) will be estimated as cost per death averted, cost per life-year saved, and cost per DALY averted. We will use recent estimates of disability weights for estimation of DALYs.[16, 17] Short-term (over study follow-up i.e. 6 months) and longer-term time horizons (extrapolated to 1, 5, and 10 years) will be used. DALYs and costs will be discounted at 3% per year, consistent with CEA guidelines (undiscounted results will also be presented). Sources of uncertainty in the results will be explored in univariate and probabilistic sensitivity analysis. [18, 19] Finally, we will compare our findings to CEA estimates for other health interventions in sub-Saharan Africa.[20, 21]

Data and Safety Monitoring

A Data Safety and Monitoring Committee (DSMC) was established at study initiation to monitor severe adverse events (SAEs) and to evaluate the statistical analysis plan and associated stopping rules. The DSMC includes expertise in clinical trials, statistics, child mortality assessment, ethics, and pediatric care in resource limited settings. Adverse events will be monitored by the DSMC. Monthly adverse event summaries will be sent to the DSMC safety officer and individual-child SAE forms, which include detailed medication history to evaluate possible drug interactions, will be sent to the safety officer per request. Each SAE will be assigned the plausibility of relatedness to study drug by study PIs. The data will not be presented by intervention group unless requested by DSMB safety officer. These reports will be descriptive (no statistical analyses). The DSMC will make recommendations regarding any imbalances in safety outcomes.

A single interim analysis for re-hospitalization-free survival will be prepared by the study statistician using O'Brien-Fleming boundaries for benefit and harm when 50% of expected person time (350 child-years) has been accrued. Assuming 157 events will be available at half of the person-time accrual, a z-score critical value of 2.797, or *p*-value < 0.005, from a Kaplan Meier log-rank test will determine the cut-off of statistical significance. A symmetric boundary will be used for benefit and harm. The DSMC will review this analysis and make a determination about study continuation. Futility will not be a basis for stopping rules because of the trials' value in understanding mechanisms of post-discharge worsening and antibiotic resistance. Assuming the

For peer review only

DSMC decides to continue the trial after the interim analysis, an alpha of 0.045 will be used as the statistical significance boundary at the final analysis.

Statistical Power

To compare rates of re-hospitalization and mortality in the 6 months following hospital discharge among Kenyan children receiving 5-day azithromycin vs. placebo. The total sample size required was calculated for the primary endpoint of time to death or hospital re-admission within the 6 month post-discharge period, assuming an alpha level of 0.05, power of 0.80, and a ratio of treatment to placebo random assignment of 1:1. In SSA, it is estimated that 2-15% of children aged less than 5 years died within 6 months of hospital discharge and 15.5% of children who survived discharge from the district hospital were re-admitted with the same diagnosis within 6-months[2, 4, 22] Assuming that an additional 5-10% of children are re-admitted for other conditions, we expect that re-hospitalizations will occur in 20.5 to 30.5% of children enrolled in the study. Combined with our expected fatality rate (2-15%), we expect the cumulative incidence of the combined endpoint to range from 22.5 to 45.5%.[22] Based on a previous trial of mass drug administration of a single dose of azithromycin in which a single dose of the antibiotic was associated with a 49% reduction in risk of death, we calculated sample sizes using estimates of reduction in risk ranging from 30-50% with the cumulative incidence range of 22.5 to 45.5%, and found the sample size required ranged from 90 to 550 children per treatment arm.[5] Using the most conservative estimates of a hazard ratio of 0.70 and 22.5% prevalence of re-admission [illegible]er arm) to achieve adequate power. We will recrui[illegible]ss to follow-up, resulting in a total planned enro[illegible]en considering mortality alone, and estimated mo[illegible]ct hazard ratios ≤0.5 for mortality rates of ≥ 8% an[illegible]

Figure 1. Power and detectable hazard ratios given a range of mortality rates

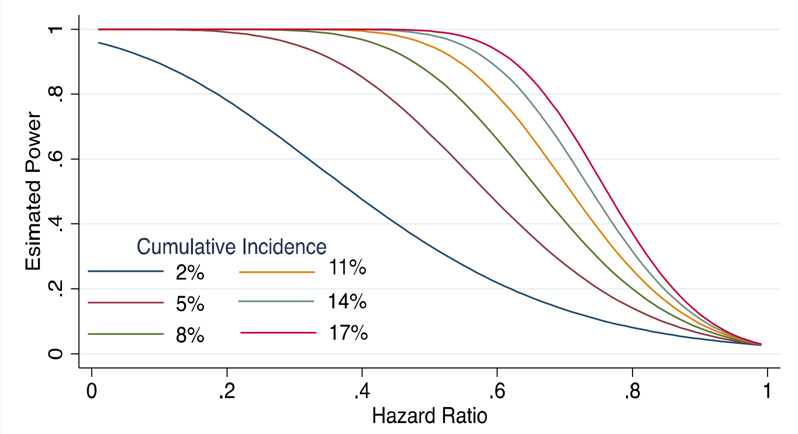

To evaluate possible mechanism(s) b[illegible]*y comparing reasons for re-hospitalization and prevalence of pathogen carriage between the randomization arms.* We calculated the minimum detectable association between treatment arm and cause-specific re-hospitalizations (hospitalization due to diarrhea, acute respiratory infection, malnutrition, or malaria vs. any other) among enrolled children, assuming an alpha level of 0.05, power of 0.80, and a ratio of treatment to placebo of 1:1. Based on data from Kenya, re-hospitalization rates due to specific causes ranged from approximately 0.5% to 5.7% in the 6 month post-discharge period.[4] By not conditioning on the child having the same diagnosis as the initial hospitalization, we expect the cumulative incidence of cause-specific re-hospitalizations to range from 2.5% to 10%. With this range of outcome rates, we will be able to detect hazard ratios of 0.48 to 0.70 for the effect of azithromycin on specific severe morbidities.

We expect 56% of children in the placebo group to have *S. pneumoniae* isolated from nasopharyngeal swabs, providing ≥80% power to detect a prevalence ratio of 0.85 (or 1.15) between the two treatment arms at each time point.[23-25] Based on prevalences of *Shigella*, *Salmonella*, *Campylobacter, Cryptosporidium, Giardia* among asymptomatic children in Western Kenya, we expect 10% of children in the placebo group to have a bacterial pathogen isolated at each time point, resulting in ≥80% power to detect differences in enteric pathogen prevalences of 0.67 (1.49) at each time point.[26]

To determine whether empiric administration of azithromycin at hospital discharge increases risk of antimicrobial resistance in commensal E. coli and pneumococcal isolates from treated children and their household contacts. We will select a random selection of 400 *E. coli* and 400 *S. pneumoniae* isolates (200 per arm) for β-lactam and macrolide resistance testing at each timepoint. We will also store all *S. pneumoniae*, *E. coli* isolates and other isolated bacteria from stool for potential future testing in the event that resistance prevalence is lower than expected. As shown in Table 3, we will have > 80% power to detect prevalence ratios

For peer review only - http://bmjopen.bmj.com/site/about/guidelines.xhtml

> 1.1, with an ability to detect the smallest effect sizes when the prevalence of resistance in the placebo group is highest.

We will enroll 300 adults in the Contact Cohort for *E. coli* and *S. pneumoniae* isolation. We expect *E.coli* to be isolated from all adults and *S.pnuemoniae* isolated from between 5-55%.[23, 27, 28] Assuming an alpha of .05, a 1:1 ratio of testable isolates, and a prevalence of resistance of 50% in the placebo arm, we will have 80% power to detect a 1.4-fold higher prevalence to 1.9-fold higher resistance prevalence in the contacts of azithromycin-treated children.

Table 3. Power (%) to detect prevalence ratios of macrolide and β-lactamase resistance in 200 *E.coli* and 200 *S. pneumoniae* isolates per treatment group

	**Resistance prevalence (%) in placebo group**						
**Resistance Prevalence (%)**	**10**	**20**	**30**	**40**	**50**	**60**	**70**
**10**							
**20**	80						
**30**	>99	64					
**40**	>99	99	55				
**50**	>99	>99	98	48			
**60**	>99	>99	>99	>99	52		
**70**	>99	>99	>99	>99	98	55	
**80**	>99	>99	>99	>99	>99	99	64

<u>*To identify correlates and intermediate markers of post-discharge mortality and hospital-readmission among hospitalized Kenyan children.*</u> Conservatively estimating a 20% loss-to-follow-up rate in the RCT and a cumulative incidence of death or re-hospitalization of 22.5%, we will have >80% power to detect hazard ratios ≥1.3 between correlates and the outcome with exposure prevalences of ≥20% or more and hazard ratios ≥1.5 for exposure prevalences <20%.

Study timeline

The trial began on June 28, 2016 and participant recruitment and follow-up will continue over a 36-month period, with anticipated final follow-up visit(s) occurring in June 2019. Primary analyses will be complete by February 2020.

Potential Challenges and Limitations

In order to ensure adequate power to detect a discernable clinically relevant difference between study groups in the primary outcome, we have combined hospital readmission with death. Preliminary studies suggest that sufficient numbers of children will reach this combined outcome. However, we have incorporated an interim analysis by the DSMB to review the accrued data and an adapted sample size could be considered if the combined event frequency is less than predicted. It is possible that since most children receive antibiotics during hospitalization, the benefit anticipated with the use of azithromycin based on previous trials of mass drug administration will not be observed. However, most hospitalized children are treated with penicillins, cephalosporins, gentamicin, or cotrimoxazole while in hospital and the broad spectrum of activity (including malaria prevention) and long half-life of azithromycin suggest that there may be additive treatment and/or prophylactic benefit. Interim analysis will allow us to determine whether children receiving specific agents during inpatient treatment are less likely to benefit and will allow us to adapt our study design, sample size, or approach if necessary. After discharge, it is difficult to ensure adherence with the full 5-day treatment course. We will measure adherence using three different measures (text message responses, bottle check boxes, and caregiver-report at follow-up visits). In addition, the mortality benefit of azithromycin observed in Ethiopia was from a single dose and in this study the first dose will be directly observed.[5] While relying on caregiver report of mortality and morbidity may lead to bias due to outcome misclassification, this misclassification should not differ between randomization arms and therefore will be non-differential. Further hospital records will be used when available to determine diagnoses. Finally, resistance prevalence may be lower than predicted, limiting power to detect clinically relevant differences in resistance prevalence between the intervention arms. We will store all isolates in the event that a greater number of isolates are needed for antimicrobial resistance testing.

Regulatory Authorities

This study has received IRB approval by the University of Washington Human Subjects Division (HSD), KEMRI Scientific and Ethics Review Unit (SERU), and the Kenya Pharmacy and Poisons Board. The clinical trial is also registered with clinicaltrials.gov (NCT02414399). Any modifications to the study protocol or consent materials will be submitted for approval all regulatory authorities before implementation. Westat® will provide external clinical, pharmacy, and laboratory monitoring.

Dissemination

For peer review only

Results of this study will be disseminated by publication in a peer-reviewed scientific journal, presented at relevant academic conferences, and amongst participating partners and health facilities in Kenya.

Author's contributions

JLW, PBP, GJS, BAR, BOS, and RN conceived of this trial and developed of this study protocol. JLW and BOS are study co-Principal Investigators and PBP is the Project Director; BAR oversaw the statistical analyses plans; JBB developed the CEA plan; KDT developed procedures for ascertaining and reporting SAEs; CJM developed procedures related to blood specimen procedures and drug adherence measurement. GJS, CJM, RN, and PBP provide scientific expertise. RLB and MA are involved in collection and management of the data. MA and PBP coordinate and oversee implementation of all clinical study procedures and SK with assistance from DR oversees all laboratory procedures. All authors contributed to the development of this manuscript and/or study procedures, and to reading and approving the final version for publication.

Funding statement

This work was funded by the National Institutes of Health Eunice Kennedy Shriver National Institute of Child Health & Human Development, grant number R01 HD079695.

Acknowledgements

Pfizer donated the Zithromax® to be used in this clinical trial, and Copan Diagnostics donated all rectal swabs and Cary-Blair media. Investigators from KEMRI-Wellcome Trust Kilifi, Jay Berkley, Anthony Scott, Joseph Waichungo, Angela Karani, Donald Akech provided microbiology expertise trainings in nasopharyngeal swab collection, STGG media preparation, and overall microbiologic quality assurance and control. Alex Awuor and Caleb Okonji, with the support of Richard Omore, provided training in anthropometric measurement. We are extremely thankful to Dr. Phillip Walson who developed azithromycin dosing regimens. Hannah Atlas and Stephanie Belanger contributed to the standard operating procedure and case report form development and implementation. Gillian Levine played an invaluable role in the proposal development.

Competing interests statement

None of the authors or study co-investigators have any competing interests to declare.

For peer review only

REFERENCES

1. Lozano R, Wang H, Foreman KJ, Rajaratnam JK, Naghavi M, Marcus JR, Dwyer-Lindgren L, Lofgren KT, Phillips D, Atkinson C *et al*: **Progress towards Millennium Development Goals 4 and 5 on maternal and child mortality: an updated systematic analysis**. *Lancet* 2011, **378**(9797):1139-1165.
2. Moisi JC, Gatakaa H, Berkley JA, Maitland K, Mturi N, Newton CR, Njuguna P, Nokes J, Ojal J, Bauni E *et al*: **Excess child mortality after discharge from hospital in Kilifi, Kenya: a retrospective cohort analysis**. *Bull World Health Organ* 2011, **89**(10):725-732, 732A.
3. Veirum JE, Sodeman M, Biai S, Hedegard K, Aaby P: **Increased mortality in the year following discharge from a paediatric ward in Bissau, Guinea-Bissau**. *Acta Paediatr* 2007, **96**(12):1832-1838.
4. Snow RW, Howard SC, Mung'Ala-Odera V, English M, Molyneux CS, Waruiru C, Mwangi I, Roberts DJ, Donnelly CA, Marsh K: **Paediatric survival and re-admission risks following hospitalization on the Kenyan coast**. *Trop Med Int Health* 2000, **5**(5):377-383.
5. Porco TC, Gebre T, Ayele B, House J, Keenan J, Zhou Z, Hong KC, Stoller N, Ray KJ, Emerson P *et al*: **Effect of mass distribution of azithromycin for trachoma control on overall mortality in Ethiopian children: a randomized trial**. *JAMA* 2009, **302**(9):962-968.
6. Keenan JD, Ayele B, Gebre T, Zerihun M, Zhou Z, House JI, Gaynor BD, Porco TC, Emerson PM, Lietman TM: **Childhood mortality in a cohort treated with mass azithromycin for trachoma**. *Clin Infect Dis* 2011, **52**(7):883-888.
7. Bwakura-Dangarembizi M. KL, Bakeera-Kitaka S., Nahirya-Ntege P., Keishanyu R., Kekitiinwa A., Bakeera-Kitaka S., Nahirya-Ntege P., Keishanyu R., Kekitiinwa A., Natukunda E., Walker S., Gibb D., Prendergast A., and ARROW Study Group: **Randomized Comparison of Stopping vs Continuing Cotrimoxazole Prophylaxis among 758 HIV+ Children on Long-term ART: The Anti-Retroviral Research for Watoto Trial**. In: *20th Conference on Retroviruses and Opportunistic Infections (CROI) 2013: March 2-6, 2013; Atlanta, Georgia*.
8. Trehan I, Goldbach HS, LaGrone LN, Meuli GJ, Wang RJ, Maleta KM, Manary MJ: **Antibiotics as part of the management of severe acute malnutrition**. *N Engl J Med* 2013, **368**(5):425-435.
9. Walker AS, Mulenga V, Ford D, Kabamba D, Sinyinza F, Kankasa C, Chintu C, Gibb DM: **The impact of daily cotrimoxazole prophylaxis and antiretroviral therapy on mortality and hospital admissions in HIV-infected Zambian children**. *Clin Infect Dis* 2007, **44**(10):1361-1367.
10. Chintu C, Bhat GJ, Walker AS, Mulenga V, Sinyinza F, Lishimpi K, Farrelly L, Kaganson N, Zumla A, Gillespie SH *et al*: **Co-trimoxazole as prophylaxis against opportunistic infections in HIV-infected Zambian children (CHAP): a double-blind randomised placebo-controlled trial**. *Lancet* 2004, **364**(9448):1865-1871.
11. O'Brien KL, Nohynek H, World Health Organization Pneumococcal Vaccine Trials Carraige Working G: **Report from a WHO working group: standard method for detecting upper respiratory carriage of Streptococcus pneumoniae**. *Pediatr Infect Dis J* 2003, **22**(2):133-140.
12. Satzke C, Turner P, Virolainen-Julkunen A, Adrian PV, Antonio M, Hare KM, Henao-Restrepo AM, Leach AJ, Klugman KP, Porter BD *et al*: **Standard method for detecting upper respiratory carriage of Streptococcus pneumoniae: updated recommendations from the World Health Organization Pneumococcal Carriage Working Group**. *Vaccine* 2013, **32**(1):165-179.
13. Serina P, Riley I, Stewart A, Flaxman AD, Lozano R, Mooney MD, Luning R, Hernandez B, Black R, Ahuja R *et al*: **A shortened verbal autopsy instrument for use in routine mortality surveillance systems**. *BMC medicine* 2015, **13**:302.
14. Serina P, Riley I, Stewart A, James SL, Flaxman AD, Lozano R, Hernandez B, Mooney MD, Luning R, Black R *et al*: **Improving performance of the Tariff Method for assigning causes of death to verbal autopsies**. *BMC medicine* 2015, **13**:291.
15. WHO**. Making choices in health: WHO guide to cost-effectiveness analysis.** 2003.
16. Murray CJ, Vos T, Lozano R, Naghavi M, Flaxman AD, Michaud C, Ezzati M, Shibuya K, Salomon JA, Abdalla S *et al*: **Disability-adjusted life years (DALYs) for 291 diseases and injuries in 21 regions, 1990-2010: a systematic analysis for the Global Burden of Disease Study 2010**. *Lancet* 2012, **380**(9859):2197-2223.

For peer review only

17. Salomon JA, Vos T, Hogan DR, Gagnon M, Naghavi M, Mokdad A, Begum N, Shah R, Karyana M, Kosen S *et al*: **Common values in assessing health outcomes from disease and injury: disability weights measurement study for the Global Burden of Disease Study 2010**. *Lancet* 2012, **380**(9859):2129-2143.
18. Gold M, Siegel JE, Russell LB, Weinstein MC**. Cost-effectiveness in health and medicine.** Oxford University Press: New York 1996.
19. Drummond MF, Torrance GW, O'Brien BJ, Stoddart GL. **Methods for the economic evaluation of healthcare programmes, 3rd edition.** Oxford University Press: New York 2005.
20. Laxminarayan R, Mills AJ, Breman JG, Measham AR, Alleyne G, Claeson M, Jha P, Musgrove P, Chow J, Shahid-Salles S *et al*: **Advancement of global health: key messages from the Disease Control Priorities Project**. *Lancet* 2006, **367**(9517):1193-1208.
21. Jamison DT, Measham A, Alleyne G, Claeson M, et al. **Disease Control Priorities in Developing Countries,** 2nd edition. Oxford University Press, 2006.
22. Wiens MO, Pawluk S, Kissoon N, Kumbakumba E, Ansermino JM, Singer J, Ndamira A, Larson C: **Pediatric post-discharge mortality in resource poor countries: a systematic review**. *PLoS One* 2013, **8**(6):e66698.
23. Abdullahi O, Karani A, Tigoi CC, Mugo D, Kungu S, Wanjiru E, Jomo J, Musyimi R, Lipsitch M, Scott JA: **The prevalence and risk factors for pneumococcal colonization of the nasopharynx among children in Kilifi District, Kenya**. *PLoS One* 2012, **7**(2):e30787.
24. Rodrigues F, Foster D, Caramelo F, Serranho P, Goncalves G, Januario L, Finn A: **Progressive changes in pneumococcal carriage in children attending daycare in Portugal after 6 years of gradual conjugate vaccine introduction show falls in most residual vaccine serotypes but no net replacement or trends in diversity**. *Vaccine* 2012, **30**(26):3951-3956.
25. Grivea IN, Tsantouli AG, Michoula AN, Syrogiannopoulos GA: **Dynamics of Streptococcus pneumoniae nasopharyngeal carriage with high heptavalent pneumococcal conjugate vaccine coverage in Central Greece**. *Vaccine* 2011, **29**(48):8882-8887.
26. Skalet AH, Cevallos V, Ayele B, Gebre T, Zhou Z, Jorgensen JH, Zerihun M, Habte D, Assefa Y, Emerson PM *et al*: **Antibiotic selection pressure and macrolide resistance in nasopharyngeal Streptococcus pneumoniae: a cluster-randomized clinical trial**. *PLoS Med* 2010, **7**(12):e1000377.
27. Adetifa IM, Antonio M, Okoromah CA, Ebruke C, Inem V, Nsekpong D, Bojang A, Adegbola RA: **Pre-vaccination nasopharyngeal pneumococcal carriage in a Nigerian population: epidemiology and population biology**. *PLoS One* 2012, **7**(1):e30548.
28. Hill PC, Akisanya A, Sankareh K, Cheung YB, Saaka M, Lahai G, Greenwood BM, Adegbola RA: **Nasopharyngeal carriage of Streptococcus pneumoniae in Gambian villagers**. *Clinical infectious diseases : an official publication of the Infectious Diseases Society of America* 2006, **43**(6):673-679.

For peer review only

SPIRIT — Standard Protocol Items: Recommendations for Interventional Trials

SPIRIT 2013 Checklist: Recommended items to address in a clinical trial protocol and related documents*

Section/item	Item No	Description	Addressed on page number
**Administrative information**			
Title	1	Descriptive title identifying the study design, population, interventions, and, if applicable, trial acronym	________
Trial registration	2a	Trial identifier and registry name. If not yet registered, name of intended registry	________
	2b	All items from the World Health Organization Trial Registration Data Set	________
Protocol version	3	Date and version identifier	________
Funding	4	Sources and types of financial, material, and other support	________
Roles and responsibilities	5a	Names, affiliations, and roles of protocol contributors	________
	5b	Name and contact information for the trial sponsor	________
	5c	Role of study sponsor and funders, if any, in study design; collection, management, analysis, and interpretation of data; writing of the report; and the decision to submit the report for publication, including whether they will have ultimate authority over any of these activities	________
	5d	Composition, roles, and responsibilities of the coordinating centre, steering committee, endpoint adjudication committee, data management team, and other individuals or groups overseeing the trial, if applicable (see Item 21a for data monitoring committee)	________

For peer review only

Introduction

Background and rationale	6a	Description of research question and justification for undertaking the trial, including summary of relevant studies (published and unpublished) examining benefits and harms for each intervention	____________
	6b	Explanation for choice of comparators	____________
Objectives	7	Specific objectives or hypotheses	____________
Trial design	8	Description of trial design including type of trial (eg, parallel group, crossover, factorial, single group), allocation ratio, and framework (eg, superiority, equivalence, noninferiority, exploratory)	____________

Methods: Participants, interventions, and outcomes

Study setting	9	Description of study settings (eg, community clinic, academic hospital) and list of countries where data will be collected. Reference to where list of study sites can be obtained	____________
Eligibility criteria	10	Inclusion and exclusion criteria for participants. If applicable, eligibility criteria for study centres and individuals who will perform the interventions (eg, surgeons, psychotherapists)	____________
Interventions	11a	Interventions for each group with sufficient detail to allow replication, including how and when they will be administered	____________
	11b	Criteria for discontinuing or modifying allocated interventions for a given trial participant (eg, drug dose change in response to harms, participant request, or improving/worsening disease)	____________
	11c	Strategies to improve adherence to intervention protocols, and any procedures for monitoring adherence (eg, drug tablet return, laboratory tests)	____________
	11d	Relevant concomitant care and interventions that are permitted or prohibited during the trial	____________
Outcomes	12	Primary, secondary, and other outcomes, including the specific measurement variable (eg, systolic blood pressure), analysis metric (eg, change from baseline, final value, time to event), method of aggregation (eg, median, proportion), and time point for each outcome. Explanation of the clinical relevance of chosen efficacy and harm outcomes is strongly recommended	____________
Participant timeline	13	Time schedule of enrolment, interventions (including any run-ins and washouts), assessments, and visits for participants. A schematic diagram is highly recommended (see Figure)	____________

For peer review only - http://bmjopen.bmj.com/site/about/guidelines.xhtml

Sample size	14	Estimated number of participants needed to achieve study objectives and how it was determined, including clinical and statistical assumptions supporting any sample size calculations	______________
Recruitment	15	Strategies for achieving adequate participant enrolment to reach target sample size	______________

Methods: Assignment of interventions (for controlled trials)

Allocation:			
Sequence generation	16a	Method of generating the allocation sequence (eg, computer-generated random numbers), and list of any factors for stratification. To reduce predictability of a random sequence, details of any planned restriction (eg, blocking) should be provided in a separate document that is unavailable to those who enrol participants or assign interventions	______________
Allocation concealment mechanism	16b	Mechanism of implementing the allocation sequence (eg, central telephone; sequentially numbered, opaque, sealed envelopes), describing any steps to conceal the sequence until interventions are assigned	______________
Implementation	16c	Who will generate the allocation sequence, who will enrol participants, and who will assign participants to interventions	______________
Blinding (masking)	17a	Who will be blinded after assignment to interventions (eg, trial participants, care providers, outcome assessors, data analysts), and how	______________
	17b	If blinded, circumstances under which unblinding is permissible, and procedure for revealing a participant's allocated intervention during the trial	______________

Methods: Data collection, management, and analysis

Data collection methods	18a	Plans for assessment and collection of outcome, baseline, and other trial data, including any related processes to promote data quality (eg, duplicate measurements, training of assessors) and a description of study instruments (eg, questionnaires, laboratory tests) along with their reliability and validity, if known. Reference to where data collection forms can be found, if not in the protocol	______________
	18b	Plans to promote participant retention and complete follow-up, including list of any outcome data to be collected for participants who discontinue or deviate from intervention protocols	______________

For peer review only - http://bmjopen.bmj.com/site/about/guidelines.xhtml

Data management	19	Plans for data entry, coding, security, and storage, including any related processes to promote data quality (eg, double data entry; range checks for data values). Reference to where details of data management procedures can be found, if not in the protocol	______________
Statistical methods	20a	Statistical methods for analysing primary and secondary outcomes. Reference to where other details of the statistical analysis plan can be found, if not in the protocol	______________
	20b	Methods for any additional analyses (eg, subgroup and adjusted analyses)	______________
	20c	Definition of analysis population relating to protocol non-adherence (eg, as randomised analysis), and any statistical methods to handle missing data (eg, multiple imputation)	______________
**Methods: Monitoring**			
Data monitoring	21a	Composition of data monitoring committee (DMC); summary of its role and reporting structure; statement of whether it is independent from the sponsor and competing interests; and reference to where further details about its charter can be found, if not in the protocol. Alternatively, an explanation of why a DMC is not needed	______________
	21b	Description of any interim analyses and stopping guidelines, including who will have access to these interim results and make the final decision to terminate the trial	______________
Harms	22	Plans for collecting, assessing, reporting, and managing solicited and spontaneously reported adverse events and other unintended effects of trial interventions or trial conduct	______________
Auditing	23	Frequency and procedures for auditing trial conduct, if any, and whether the process will be independent from investigators and the sponsor	______________
**Ethics and dissemination**			
Research ethics approval	24	Plans for seeking research ethics committee/institutional review board (REC/IRB) approval	______________
Protocol amendments	25	Plans for communicating important protocol modifications (eg, changes to eligibility criteria, outcomes, analyses) to relevant parties (eg, investigators, REC/IRBs, trial participants, trial registries, journals, regulators)	______________

For peer review only - http://bmjopen.bmj.com/site/about/guidelines.xhtml

Consent or assent	26a	Who will obtain informed consent or assent from potential trial participants or authorised surrogates, and how (see Item 32)	_______________
	26b	Additional consent provisions for collection and use of participant data and biological specimens in ancillary studies, if applicable	_______________
Confidentiality	27	How personal information about potential and enrolled participants will be collected, shared, and maintained in order to protect confidentiality before, during, and after the trial	_______________
Declaration of interests	28	Financial and other competing interests for principal investigators for the overall trial and each study site	_______________
Access to data	29	Statement of who will have access to the final trial dataset, and disclosure of contractual agreements that limit such access for investigators	_______________
Ancillary and post-trial care	30	Provisions, if any, for ancillary and post-trial care, and for compensation to those who suffer harm from trial participation	_______________
Dissemination policy	31a	Plans for investigators and sponsor to communicate trial results to participants, healthcare professionals, the public, and other relevant groups (eg, via publication, reporting in results databases, or other data sharing arrangements), including any publication restrictions	_______________
	31b	Authorship eligibility guidelines and any intended use of professional writers	_______________
	31c	Plans, if any, for granting public access to the full protocol, participant-level dataset, and statistical code	_______________
**Appendices**			
Informed consent materials	32	Model consent form and other related documentation given to participants and authorised surrogates	_______________
Biological specimens	33	Plans for collection, laboratory evaluation, and storage of biological specimens for genetic or molecular analysis in the current trial and for future use in ancillary studies, if applicable	_______________

*It is strongly recommended that this checklist be read in conjunction with the SPIRIT 2013 Explanation & Elaboration for important clarification on the items. Amendments to the protocol should be tracked and dated. The SPIRIT checklist is copyrighted by the SPIRIT Group under the Creative Commons "Attribution-NonCommercial-NoDerivs 3.0 Unported" license.

For peer review only - http://bmjopen.bmj.com/site/about/guidelines.xhtml

BMJ Open

Azithromycin to prevent post-discharge morbidity and mortality in Kenyan children: A protocol for a randomized, double-blind, placebo-controlled trial (the Toto Bora trial)

Journal:	*BMJ Open*
Manuscript ID	bmjopen-2017-019170.R1
Article Type:	Protocol
Date Submitted by the Author:	09-Oct-2017
Complete List of Authors:	Pavlinac, Patricia; University of Washington, Global Health<br>Singa, Benson; Kenya Medical Research Institute; Childhood Acute Illness and Nutrition Network<br>John-Stewart, G; University of Washington, Global Health, Epidemiology, Pediatrics, Allergy and Infectious Disease<br>Richardson, BA; University of Washington, Global Health, BIostatistics<br>Brander, Rebecca; University of Washington, Epidemiology<br>McGrath, Christine; University of Washington, Global Health<br>Tickell, Kirkby; University of Washington, Global Health; Childhood Acute Illness and Nutrition Network<br>Amondi, Mary; Kenya Medical Research Institute<br>Rwigi, Doreen; Kenya Medical Research Institute<br>Babigumira, Joseph; University of Washington, Global Health, Pharmacy<br>Kariuki, Sam; Kenya Medical Research Institute<br>Nduati, Ruth; University of Nairobi<br>Walson, Judd; University of Washington, Department of Global Health
**Primary Subject Heading**:	Global health
Secondary Subject Heading:	Paediatrics, Infectious diseases
Keywords:	Child mortality, Post-discharge interventions, Toto Bora Trial, Targeted empiric antibiotic therapy

SCHOLARONE™
Manuscripts

Title: Azithromycin to prevent post-discharge morbidity and mortality in Kenyan children: A protocol for a randomized, double-blind, placebo-controlled trial (the Toto Bora trial)

Running head: AZM to prevent post-discharge morbidity and mortality

Authors: Pavlinac PB[1], Singa BO[7,8], John-Stewart GC[1-4], Richardson BA[1,5], Brander RL[2], McGrath CJ[1], Tickell KD[1,8], Amondi M[7], Rwigi D[7], Babigumira JB[1,6], Kariuki S[7], Nduati R[9], Walson JL[1-4,8]

Affiliations: Departments of [1]Global Health, [2]Epidemiology, [3]Pediatrics, [4]Allergy and Infectious Disease, [5]Biostatistics, [6]Pharmacy, University of Washington, Seattle, WA, USA; [7]Kenya Medical Research Institute, Nairobi, Kenya; [8]The Childhood Acute Illness & Nutrition Network, Nairobi, Kenya; [9] University of Nairobi, Nairobi, Kenya

Correspondence: Patricia Pavlinac, 325 9th Ave, Box 359931, Seattle, WA 98104 (ppav@uw.edu)

Sponsored by: This trial is sponsored by the National Institutes of Health Eunice Kennedy Shriver National Institute of Child Health & Human Development (R01 HD079695) and drug donation by Pfizer IIR #WI201906. JLW is supported by the Childhood Acute Illness and Nutrition Network (CHAIN, OPP1131320). GJS is supported by a National Institute of Health mentoring award (grant number K24-HD054314) and PBP and GJS are supported by the International Core of the University of Washington Center for AIDS Research (CFAR; Seattle, WA, USA), an NIH funded program (P30 AI027757).

Word count: 5,315 (excluding tables and figures)

For peer review only

ABSTRACT

Introduction: Child mortality due to infectious diseases remains unacceptably high in much of sub-Saharan Africa. Children who are hospitalized represent an accessible population at particularly high-risk of death, both during and following hospitalization. Hospital discharge may be a critical time point at which targeted use of antibiotics could reduce morbidity and mortality in high-risk children.

Methods and analysis: In this randomized, double-blind, placebo-controlled trial (Toto Bora), 1400 children aged 1 to 59 months discharged from 2 hospitals in western Kenya, in Kisii and Homa Bay, will be randomized to either a 5-day course of azithromycin or placebo to determine whether a short-course of azithromycin reduces rates of re-hospitalization and/or death in the subsequent 6-month period. The primary analysis will be modified intention-to-treat and will compare the rates of re-hospitalization or death in children treated with azithromycin or placebo using Cox proportional hazard regression. The trial will also evaluate the effect of a short course of azithromycin on enteric and nasopharyngeal infections and cause-specific morbidities. We will also identify risk factors for post-discharge morbidity and mortality and subpopulations most likely to benefit from post-discharge antibiotic use. Antibiotic resistance in *Escherichia coli* and *Streptococcus pneumoniae* among enrolled children and their primary caregivers will also be assessed and cost-effectiveness analyses performed to inform policy decisions.

Ethics and dissemination: Study procedures were reviewed and approved by the institutional review boards of the Kenya Medical Research Institute, the University of Washington, and the Kenyan Pharmacy and Poisons Board. The study is being externally monitored and a data safety and monitoring committee has been assembled to monitor patient safety and to evaluate the efficacy of the intervention. The results of this trial will be published in peer-reviewed scientific journals and presented at relevant academic conferences and to key stakeholders.

Trial registration number: NCT02414399

Key words: child mortality, Toto Bora, targeted empiric antibiotic therapy, post-discharge interventions

STRENGTHS AND LIMITATIONS OF THIS STUDY

Strengths

- Randomized, placebo-controlled, double-blinded design and modified intention-to-treat analysis will ensure unbiased treatment effect measure
- Comprehensive data are collected, including biological specimens for all child participants and a subset of adult caregivers, for analyses of mechanisms of post-discharge morbidity and mortality, subsets of children most likely to benefit from the antibiotic, as well as assessments of antibiotic resistance and cost-effectiveness
- Results will likely be generalizable due to the limited exclusion criteria, large sample size, and multiple study sites
-

Limitations

- Causes of death and re-hospitalization may be misclassified due to limited availability of medical records and recall bias in caregiver report
- The primary endpoint of this study is a combined outcome of re-hospitalization and death, which, while improving statistical power, may present challenges for interpretation
- Children in both intervention arms may receive other antibiotics over the course of follow-up

For peer review only

BACKGROUND

An estimated 3.5 million deaths occur annually in children less than 5 years of age in sub-Saharan Africa (SSA), approximately 70% of which are due to infectious causes. Children who were recently hospitalized have mortality rates 6 to 8-fold higher than similarly-aged children from the same community.[1-3] Post-discharge mortality rates as high as 15% have been documented in the 12 months following discharge, with mortality risk remaining elevated up to two years post-discharge.[4-8] Children who are very young, malnourished, or HIV-infected are at particularly high risk of post-discharge mortality within the 3 months following discharge. [1-4 6-8] Children being discharged from hospital in SSA may represent an accessible high-risk population in which to target interventions to reduce mortality and morbidity.

Targeted antibiotic interventions, including the use of cotrimoxazole among HIV-infected children and the use of amoxicillin or cefdinir among children with severe acute malnutrition (SAM), have been shown to reduce morbidity and mortality in these specific vulnerable populations.[9-12] Other trials of targeted antibiotic use in vulnerable populations, including cotrimoxazole in HIV-exposed uninfected (HEU) children and in children with SAM, have failed to demonstrate a mortality benefit.[13 14] In contrast, non-targeted mass drug administration of a single dose of azithromycin halved mortality rates among Ethiopian children living in communities randomized to receive the antibiotic.[15 16] Concerns about the potential emergence of antibiotic resistance, possible toxicity, and feasibility of delivery are barriers to the non-targeted antibiotic distribution strategies.

A short-course of azithromycin given to children with recent severe illness being discharged from hospital may optimize benefit while reducing both individual and population level risks. Azithromycin may reduce post-discharge morbidity and mortality through infection related mechanisms such as treating undiagnosed, incompletely treated or nosocomial infections or by protecting against new or recrudescent infections that occur during recovery. Azithromycin may also act through non antimicrobial pathways such as by anti-inflammatory and/or immunomodulatory effects.

OBJECTIVE

The primary objective of this double-blind, placebo-controlled, randomized controlled trial (RCT) is to determine whether a 5-day course of azithromycin in children age 1 to 59 months discharged from hospital in western Kenya reduces rates of re-hospitalizations and/or death in the subsequent six months. The secondary objectives are (1) to evaluate possible mechanism(s) by which azithromycin may affect morbidity and mortality, by comparing reasons for re-hospitalization and prevalence of enteric and nasopharyngeal infections between the randomization arms; (2) to determine whether empiric administration of azithromycin at hospital discharge increases risk of antibiotic resistance in commensal *Escherichia coli* (*E. coli*) and *Streptococcus pneumoniae (S. pneumoniae)* isolates from treated children and their primary caregiver; (3) to identify correlates and intermediate markers of post-discharge mortality and hospital readmission; (4) to determine the cost-effectiveness of post-discharge administration of a 5-day course of azithromycin in settings of varying antibiotic use, re-hospitalization rates, and mortality rates; and (5) to create a repository of stool, nasopharyngeal, and blood specimens from highly characterized, recently discharged children, half of whom are treated with azithromycin, to be used to address future research questions.

METHODS

Reporting of this study protocol has been verified in accordance with the SPIRIT (Standard Protocol Items for Randomized Trials) recommendations.

Eligibility

Children age 1 to 59 months old weighing at least 2 kg who have been hospitalized, and subsequently discharged, will be eligible for inclusion. Caregivers of potentially eligible children must be at least 18 years of age or classified as an emancipated minor and be willing to participate in the Contact Cohort if randomly selected. Children will be excluded if: azithromycin is contraindicated (children taking or prescribed other macrolide antibiotics, such as erythromycin or clarithromycin, or the protease inhibitor, lopinavir); they were admitted to hospital for a trauma, injury, or a birth defect; they do not plan to remain in the study site catchment area for at least 6 months; the legal guardian does not provide consent; or if a sibling was enrolled in the trial on the same day of discharge.

For peer review only - http://bmjopen.bmj.com/site/about/guidelines.xhtml

Recruitment

Children will be recruited from the inpatient wards of Kisii Teaching & Referral and Homa Bay County Hospitals where study staff will accompany hospital staff on ward rounds to identify children being discharged each day. All discharged children, as determined by the onsite hospital clinicians, will be screened by study staff during working hours. If the caregiver is interested in participating and indicates consent for screening, the study staff will screen the child for eligibility, and if eligible, will obtain informed consent for study participation. Informed consent includes an explanation of the potential risks and benefits of the study and additional provision for use of participant data and samples for future studies, and will be conducted in the language of the respondent's choosing (English, Kiswahili, Kisii, or Luo). The parent or guardian (primary caregiver) must sign written informed consent (or provide a witnessed thumbprint if not literate) prior to enrollment.

Enrollment

Children will be enrolled at the time of discharge by the clinical staff. At enrollment, primary caregivers will be interviewed to assess demographic information, medical history, and detailed contact information for the child (Table 1). Medical records will also be used to abstract information from the hospitalization (including presenting diagnosis, medical management, length of stay, procedures performed, relevant medical history, physical examination, and laboratory data). All enrolled participants will undergo a physical examination performed by the study clinician, including measurement of height (in children ≥ 24 months), length (in children <24 months), weight, and mid-upper arm circumference (MUAC), each of which will be measured three times. HIV status will be obtained from medical records or from performed testing if records are not available. Detailed home location and contact information will be collected to enable patient tracing.

Table 1. Summary of data collected among enrolled children at each study visit

Enrollment visit (hospital discharge)	3 month follow up visit	6 month follow up visit	Unscheduled visits
• Questionnaire of sociodemographic, clinical history, treatments prescribed in hospital and at discharge, hospitalization costs, dietary factors, household factors, and environmental exposures<br>• Physical exam<br>• Anthropometry<br>• Abstraction of medical records (if re-hospitalized)<br>• Verbal autopsy (or abstracted medical records)<br>• Heel/finger prick (HIV and malaria)<br>• Stool collection (*Shigella*, *Salmonella*, *Campylobacter*, *Escherichia coli*, *Cryptosporidium*, and Giardia)<br>• Nasopharyngeal swab collection (*Streptococcus pneumoniae*)	• Questionnaire of study drug administration, and reported illnesses, hospitalization costs, change in clinical history, and treatments since last visit<br>• Physical exam<br>• Anthropometry<br>• Abstraction of medical records (if re-hospitalized)<br>• Verbal autopsy (or abstracted medical records)<br>• Heel/finger prick (HIV and malaria)<br>• Stool collection (*Shigella*, *Salmonella*, *Campylobacter*, *Escherichia coli*, *Cryptosporidium*, and Giardia)<br>• Nasopharyngeal swab collection (*Streptococcus pneumoniae*)	• Questionnaire of reported illnesses, hospitalization costs, change in clinical history, and treatments since last visit<br>• Physical exam<br>• Anthropometry<br>• Abstraction of medical records (if re-hospitalized)<br>• Verbal autopsy (or abstracted medical records)<br>• Heel/finger prick (HIV and malaria, sickle-cell)<br>• Stool collection (*Shigella*, *Salmonella*, *Campylobacter*, *Escherichia coli*, *Cryptosporidium*, and Giardia)<br>• Nasopharyngeal swab collection (*Streptococcus pneumoniae*)	• Questionnaire of reported illnesses since last scheduled visit, change in clinical history, and treatments since last visit<br>• Abstraction of medical records (if re-hospitalized)<br>• Verbal autopsy (or abstracted medical records)

Specimen collection

Specimens will be collected at enrollment (prior to study medication administration, as well as at 3 and 6-month follow-up visits). All children will also be asked to provide a whole stool for enteric pathogen identification and storage. Stool samples will be divided within one hour of collection for the following purposes: 1) placed in Cary-Blair for eventual bacterial culture (FecalSwab Cary-Blair Collection and Transport Systemt^{TM}, Copan Diagnostics), 2) immediately tested for *Giardia* and *Cryptosporidium* using the immunoassay (Quik Chek^{TM}, Alere) and 3) placed in -80°C storage for future molecular determination of pathogen or commensal flora and markers of gut function (whole stool for these purposes will be divided into two separate vials). If a child cannot produce whole stool, two flocked rectal swabs (Pedatric FLOQswab^{TM},

For peer review only

Copan Diagnostics) will be collected and one placed in Cary-Blair and the other stored in -80°C for future analyses.

One flocked dry nasopharyngeal swab (Copan Diagnostics) will also be collected from all enrolled children at each time point, immediately placed in skim milk, tryptone, glucose, and glycerine (STGG) media, and frozen (-80°C) within 1 hour of collection for future *S.pnuemoniae* culture.[17 18] Primary caregivers in the Contact Cohort will also be asked to provide a stool sample (or 2 rectal swabs) and nasopharyngeal sample at each visit for testing and storage as described above.

Venous blood (up to 1 teaspoon [5mL]) will be collected from all enrolled children and caregivers enrolled in the Contact Cohort at each at each time point into EDTA tube and separated for the following purposes: 1) 0.5mL for immediate HIV-testing (if indicated according to Kenyan Ministry of Health guidelines), 2) 0.4mL for a thin malaria smear which will be stored at room temperature, 3) 0.4mL for a dried blood spot, and 4) 2-4mL for plasma and buffy coat isolation and -80°C storage.

Randomization

Block randomization (1:1) in random sized blocks of no more than 10, stratified by site, will be used. Primary randomization will include allocation to the Contact Cohorts at a ratio of 1:5 (resulting in 150 per treatment arm). Each subject will be assigned a Patient Identification Number (PID), and the randomization code linking each PID to the allocated treatment will be generated by a designated statistician and maintained by the University of Washington Research Pharmacy. Study participants, investigators (other than the statistician), the study staff, hospital clinicians, and persons involved in data management or analysis will remain blinded to the allocation group during all data collection phases of the study.

Intervention

Caregivers of enrolled children will be provided a 5-day course of oral suspension formulation azithromycin (Zithromax® from Pfizer, 10 mg/kg on day 1, followed by 5mg/kg/day on days 2-5) or identically appearing and tasting placebo at discharge. Identically appearing bottles will be pre-labelled with the PID. Dosing ranges were determined such that a given child would never be under-dosed or over-dosed by more than 20% of the weight-specific intended dose (Table 2). The day 1 dose will be split in half and the first half administered by the study clinician (to be observed by the caregiver) followed by the second half administered by the caregiver under careful observation of the study staff. Caregivers will be provided with visual instructions in the language of their choosing (English, Kiswahili, Luo, Kisii).

Table 2. Azithromycin dosing chart by child weight

Weight (kg)	Day 1 dose (mL)	Day 2-5 dose (mL)
**2.0**	0.25 x 2	0.25
**2.1-2.4**	0.30 x 2	0.30
**2.5-2.8**	0.35 x 2	0.35
**2.9-3.2**	0.40 x 2	0.40
**3.3-3.6**	0.45 x 2	0.45
**3.7-4.0**	0.50 x 2	0.50
**4.1-4.8**	0.60 x 2	0.6
**4.9-5.6**	0.70 x 2	0.7
**5.7-6.8**	0.85 x 2	0.85
**6.9-8.0**	1.0 x 2	1.0
**8.1-9.6**	1.2 x 2	1.2
**9.7-11.2**	1.4 x 2	1.4
**11.3-13.6**	1.6 x 2	1.6
**13.7-16.0**	2.0 x 2	2.0
**16.1-19.2**	2.4 x 2	2.4
**19.3-23.2**	2.9 x 2	2.9
**23.3-25.0**	3.2 x 2	3.2

Automated daily text message drug administration reminders will be sent for the four days following discharge and caregivers asked to respond with whether or not the child took the daily dose. The response text message will be free of charge to caregivers and caregivers will be reimbursed for each response at the final study visit. Caregivers are also asked to record each administered dose on the bottle and to return bottles at the 3 month follow-up visit.

Follow-up Procedures

All enrolled children and primary caregivers will be scheduled to return to the health facility at 3 and 6 months following enrollment to collect clinical information and samples. Anthropometric measurements will be obtained from all children and caregivers at both follow up visits (height/length, weight, MUAC) and caregivers will be asked about any hospitalizations occurring since the last time the child was seen by study staff. Caregivers will be provided with 400KSH (approximately $4USD) to cover the cost of their round-trip transportation.

Transportation cost will be reimbursed at each follow-up visit. If the participant does not return at their scheduled time,

For peer review only

study staff will attempt to make contact with the primary caregiver via cell phone; if no telephone number is provided, or if the participant cannot be reached, study staff will trace the child to the household within 2 weeks of the scheduled follow-up time.

During scheduled follow-up visits, study staff will use a standardized questionnaire to ascertain history of recent illness/morbidity, post-discharge medication use including antibiotic treatment, and current condition of the child (any hospitalizations, admission and discharge date of any hospitalization, vital status, date of death if applicable). If caregivers report a hospitalization, causes of admission, medication administration, cost of admission, and length of stay will be ascertained from both caregivers and medical records, when available.

Caregivers will be encouraged, at enrollment and at each subsequent contact, to bring the child to the study health facility at any time the child is sick. Study staff will triage children to the appropriate health facility staff and will conduct a brief unscheduled visit questionnaire to ascertain adverse event information. If the unscheduled visit leads to a hospitalization, this will trigger the completion of a hospital admission form.

If at any point during follow-up a child dies, a verbal autopsy using the Population Health Metrics Research Consortium Shortened Verbal Autopsy Questionnaire.[19] If the death occurred in a hospital, data from the hospital records, including cause of death, if available, will be abstracted. If a death certificate is available, cause(s) and timing of death will be abstracted.

Final causes of re-hospitalization and death will be determined after data collection is complete by an independent adjudication committee comprised of clinicians specializing in pediatrics and infectious disease. Sources of cause of re-hospitalization (medical records and caregiver report) and causes of death (causes automatically assigned from the verbal autopsy using SmartVA-Analyze [Tariff 2.0 Method][20], hospital records, or death certificates) will be presented to the adjudication committee for final cause assignment.

Laboratory Procedures

Stool (rectal swabs), nasopharyngeal swabs and blood will be collected as described above and undergo either immediate or future laboratory testing as described in Table 3. All biological samples will be collected by staff trained in biosafety and Good Clinical Laboratory Practice (GCLP). Samples will be processed in Kenya when technology is available at the Kenya Medical Research Institute (KEMRI) Wellcome Trust or Centre for Microbiology Research [CMR]. Metagenomic analyses and/or analyses that require technology not available in Kenya will be performed at the University of Washington. If stool culture results report *Shigella* or *Salmonella* infection, the study staff will contact the child's caregiver and encourage the caregiver to bring the child back for an evaluation and potential treatment if the child is symptomatic.

Table 3. Sample storage and processing descriptions

Specimen Collected	Purpose	Tests Performed
Stool/ flocked rectal swabs	Bacterial ID and storage for AST	Fresh samples/rectal swabs will be cultured to identify *Shigella*, *Salmonella*, *Campylobacter*, and *Escherichia coli (E.coli)* using standard microbiologic methods and biochemically confirmed using bioMérieux's API® strips. All *Shigella*, *Salmonella*, and *Campylobacter* isolates, as well as a random subset of *E.coli* isolates will undergo antibiotic resistance testing using disc diffusion for the following antibiotics: amoxicillin-clavulanic acid (augmentin), ampicillin, azithromycin, chloramphenicol, ciprofloxacin, ceftriaxone, ceftazidime, cefoxitin, gentamicin, imipenem, trimethoprim-sulfamethoxazole, ceftazidime/clavulanate (ESBL), cefotaxime/clavulanate (ESBL). Categorizations of susceptible, intermediate, and resistant will be determined using zone-size cut-offs outlined in Clinical and Laboratory Standards Institute (CLSI) interpretive standards.
	Parasite detection	Fresh stool and rectal swabs will be tested for *Giardia* and *Cryptosporidium* using the immunoassay Giardia/ Cryptosporidium QUIK CHEK™.
	Storage	Stool/ flocked swabs and colonies of *E.coli*, *Shigella* spp., *Salmonella* spp., and *Campylobacter* spp. will be stored at -80°C.
Nasopharyngeal Swabs	Bacterial isolation, storage, and resistance testing	*Streptococcus pneumoniae* (*S. pneumoniae*) colonies will be isolated using standard microbiologic or molecular diagnostic protocols and susceptibility testing performed using standard microbiologic or molecular techniques. A random subset of *S. pneumoniae* isolates will undergo antimicrobial resistance testing using disc diffusion for the following antibiotics: amoxicillin-clavulanic acid (Augmentin), ampicillin, azithromycin, chloramphenicol, ciprofloxacin, ceftriaxone, ceftazidime, cefoxitin, gentamicin, imipenem, and trimethoprim-sulfamethoxazole. Categorizations of susceptible, intermediate, and resistant will be determined using zone-size cut-offs outlined in CLSI interpretive standards.
	Storage	Back-up sample and *S.pneumoniae* colonies will be will be stored at -80°C.

For peer review only

Blood	HIV and malaria testing	HIV testing will be performed per Kenyan National Guidelines and malaria microscopy performed using standard methods.
	Storage	Plasma and buffy coat will be stored at -80°C. Dried blood spots will be stored at room temperature.

Data Management and Confidentiality

Personal information about the participants, including medical records and data ascertained per caregiver interview, will be securely stored in files in the study offices at the study sites. Only pre-designated study staff will have access to the files. Data will be entered into an electronic database (Dacima® Electronic Data Capture) regularly by study staff. Access to the electronic database will be secured using password protected accounts for study staff. Data reports of screening, enrollment, and exclusion totals will be disseminated to the study team on a weekly basis; reports including baseline demographic characteristics, laboratory results, adherence data, and serious adverse event summaries will be distributed to study co-investigators and data monitors quarterly. Data will be regularly queried to facilitate ongoing data cleaning.

Data Analysis

Primary endpoints:
The primary study endpoint is a combined outcome of mortality and hospital readmission, as re-hospitalization is highly associated with risk of subsequent poor outcome. Re-hospitalizations that are a continuation of management from the previous hospitalization (such as elective blood transfusion) or that occur during enrollment procedures, due to a clinical deterioration post-discharge, will be excluded from the analysis. Loss to follow-up will be defined as non-attendance at both follow-up visits despite one month of active tracing and no clear evidence of death.

Secondary endpoints include:

1. *Cause-specific re-hospitalizations* assessed by questionnaire (maternal recall of diagnosis) at month 3 and month 6 follow-up visits and medical record review (discharge diagnosis). In cases when both sources are available, information from the medical record will be considered as the primary source. Separate analyses will be performed for each diagnosis: diarrhea, acute respiratory infection, malnutrition, or malaria.
2. *Mild, moderate, and severe events* that did not result in re-hospitalization, including diarrhea, vomiting, skin rash, lip swelling, difficulty breathing/wheeze, and seizure will be ascertained by caregivers identified by the study clinicians during clinical exams at scheduled follow-up visits or during unscheduled visits. Severity (grade 1-3) will be defined according to 2014 Division of AIDS (DAIDS) Table for Grading the Severity of Adult and Pediatric Adverse Events.
3. *Enteric pathogen carriage*, operationalized as presence of a bacterial pathogen-*Shigella* species (spp.), *Campylobacter* spp., or *Salmonella* spp., or parasite- *Giardia* or *Cryptosporidium* in stool or rectal swabs assessed at month 3 and month 6 follow-up visits.
4. *Streptococcus pneumoniae (S. pneumoniae)* isolated from nasopharyngeal swab cultures at month 3 and month 6 follow-up visits.
5. *Antibiotic resistance*, specifically resistance to azithromycin, ampicillin, augmentin, ciprfrofloxacin, trimethoprim-sulfamethoxazole, in *E.coli* and *S. pneumoniae* isolates, and presence of ESBL in *E.coli* isolates, from month 3 and month 6 follow-up visits.

To compare rates of re-hospitalization and mortality in the 6 months following hospital discharge among Kenyan children receiving 5-day azithromycin vs. placebo. Primary analyses will be modified intent-to-treat (mITT) based on randomization allocation to the 5-day course of azithromycin versus placebo. Cumulative incidence of death or first re-hospitalization will be compared between treatment groups using Cox proportional hazards regression. Participants will be censored at the date of their first re-hospitalization, or at the date of death. Median time to hospitalization-free survival will be compared between randomization groups using Kaplan-Meier (K-M) survival analysis and associated log-rank test. If the baseline assessment of randomization reveals an imbalance in characteristics between the treatment groups, we will evaluate these variables as potential confounders in a sub-analysis secondary to the mITT. Potential baseline confounders will be added stepwise in a multivariable Cox model and maintained in the model if adjustment changes the hazard

For peer review only

ratio by more than 10%. In per protocol analyses also secondary to the mITT, we will compare treatment effects in groups defined by self-reported adherence to the 5-day course of azithromycin (5 doses vs. < 5 doses; ≥3 doses vs. <3 doses; >1 dose vs. 1 dose only). In addition, we will conduct Cox regression and K-M survival analyses for time to mortality and time to re-hospitalization as separate endpoints to understand intervention effects on these outcomes individually. The assumption of proportional hazards will be checked in all models using graphical methods including plotting a ln(-ln(S(t))) plot for each treatment group and assessing the parallelism of the two lines and by plotting Schoenfeld residuals over time. If there is substantial missing covariate data, multiple imputation using the Markov chain Monte Carlo (MCMC) method will be used to impute covariate information. Missing outcome data (death or re-hospitalization) will not be imputed, but participants will be censored at the last follow-up visit therefore contributing some person-time to the analysis. In sensitivity analyses, we will compare treatment effects in children whose caregivers report no additional antibiotic use over follow-up and separately, who report no additional azithromycin use specifically, and in subsets of children defined by age, site, and discharge diagnosis.

To evaluate possible mechanism(s) by which azithromycin may affect morbidity and mortality, by comparing reasons for re-hospitalization and change in prevalence of pathogen carriage between the randomization arms. To evaluate the association between azithromycin and the rates of cause-specific re-hospitalizations (hospitalization due to diarrhea, acute respiratory infection, malnutrition, or malaria) we will use Anderson-Gill proportional hazards modeling with previous re-hospitalizations included as time-dependent covariates in the model to capture the dependent structure of recurrence times. Because we will not have granularity in the time points other than 3 months and 6 months for assessment of pathogen carriage, we will compare the prevalence of a bacterial and parasitic pathogens (*Shigella*, *Salmonella*, *Campylobacter, Cryptosporidium, Giardia*) at 3 and 6 months by randomization arm using generalized estimating equations (GEE) with a Poisson link, exchangeable correlation structure, and will adjust for baseline presence of a bacterial pathogen. To determine whether an observed association between the intervention and pathogen carriage wanes over time, we will test the hypothesis that the prevalence ratios comparing carriage in intervention arms are the same at the two follow-up time points using a chi-squared test.

To determine whether empiric administration of azithromycin at hospital discharge increases risk of antibiotic resistance in commensal E. coli and pneumococcal isolates from treated children and their household contacts. Among children and adult household contacts in whom commensal *E. coli* and/or *S. pneumoniae* are isolated, we will compare the proportion of isolates resistant to azithromycin, ampicillin, augmentin, ciprofloxacin, and trimethoprim-sulfamethoxazole, between randomization arms and Contact Cohorts for each arm, at 3 and 6 months using GEE with a Poisson link and exchangeable correlation structure. A chi-squared test will be used to determine whether the association between intervention arm and antibiotic resistance wanes over time. Because the likelihood of having a bacterial pathogen isolated may depend on baseline factors, including intervention arm, we will conduct secondary analyses utilizing propensity scores to account for the potential differential likelihood of having antibiotic susceptibility testing performed, which will allow us to make inference to the entire study population and their contacts. Also we will compare resistance proportions among children (as opposed to among isolates) where absence of an isolated bacteria is considered not resistant.

To identify correlates and intermediate markers of post-discharge mortality and hospital-readmission among hospitalized children. Enrollment hospital admission diagnosis, indicators of malnutrition, age, HIV-exposure and HIV-infection status, sickle cell anemia, and randomization arm will be assessed in a multivariable Cox regression model to identify correlates of the primary endpoint of death and/or hospital-readmission independent of the treatment effect. In addition, Cox regression models will also be built for correlates of mortality and correlates of re-admission individually to understand distinct cofactors for each of these outcomes.

To determine the cost-effectiveness of post-discharge administration of a 5-day course of azithromycin in settings of varying antibiotic use, re-hospitalization rates, and mortality rates. Costs analysis: We will assess the costs of all supplies, services and equipment necessary to implement the intervention (direct medical costs). The perspective will be that of the healthcare provider, i.e. Kenya's Ministry of Health. Using WHO guidelines and its ingredients approach, we will quantify the resources and associated unit costs required to deliver a 5-day course of azithromycin, organized in standard expenditure categories: personnel (salaries), supplies including drugs, equipment, services, space and overhead. We will also measure the costs of severe child hospitalizations, the costs for the different types of personnel employed (e.g. nurses/doctors) and the time

For peer review only - http://bmjopen.bmj.com/site/about/guidelines.xhtml

demanded from them for conducting the intervention.[21] When data are missing, they will be complemented by data extracted from the literature and other available sources. Full incremental costs will be derived, with estimation of the potential healthcare cost-offset realized in avoiding severe hospitalizations. Costs will be measured in local currency (Kenyan Shilling) and converted into US$. Our main metric will be cost per child treated. Cost-effectiveness analysis (CEA): we will develop a CEA mathematical model, and estimate incremental costs and cost-effectiveness for implementation of the intervention. The model will include two components: costs (described immediately above) and health benefits. The study will provide clinical outcomes (mortality/morbidity) over a 6-month follow-up period. Subsequently, deaths averted, life-years saved and disability-adjusted life years (DALYs) averted by the intervention will be estimated. We will estimate: a) incremental costs and b) incremental cost-effectiveness of the intervention vs. status quo. *Incremental costs* are the net sum of the costs to implement the intervention compared with status quo, and the costs averted due to the decrease in severe child hospitalizations. *Incremental cost-effectiveness* ratios (ICERs) will be estimated as cost per death averted, cost per life-year saved, and cost per DALY averted. We will use recent estimates of disability weights for estimation of DALYs.[22 23] Short-term (over study follow-up i.e. 6 months) and longer-term time horizons (extrapolated to 1, 5, and 10 years) will be used. DALYs and costs will be discounted at 3% per year, consistent with CEA guidelines (undiscounted results will also be presented). Sources of uncertainty in the results will be explored in univariate and probabilistic sensitivity analysis. [24 25] Finally, we will compare our findings to CEA estimates for other health interventions in sub-Saharan Africa.[26 27]

Data and Safety Monitoring

A Data Safety and Monitoring Committee (DSMC) will be established at study initiation to monitor severe adverse events (SAEs) and to evaluate the statistical analysis plan and associated stopping rules. The DSMC includes expertise in clinical trials, statistics, child mortality assessment, ethics, and pediatric care in resource limited settings. Adverse events will be monitored by the DSMC. Monthly adverse event summaries will be sent to the DSMC safety officer and individual-child SAE forms, which include detailed medication history to evaluate possible drug interactions, will be sent to the safety officer per request. Each SAE will be assigned the plausibility of relatedness to study drug by study PIs. The data will not be presented by intervention group unless requested by DSMB safety officer. These reports will be descriptive (no statistical analyses). The DSMC will make recommendations regarding any imbalances in safety outcomes.

A single interim analysis for re-hospitalization-free survival will be prepared by the study statistician using O'Brien-Fleming boundaries for benefit and harm when 50% of expected person time (350 child-years) has been accrued. Assuming 157 events will be available at half of the person-time accrual, a z-score critical value of 2.797, or *p*-value < 0.005, from a Kaplan Meier log-rank test will determine the cut-off of statistical significance. A symmetric boundary will be used for benefit and harm. The DSMC will review this analysis and make a determination about study continuation. Futility will not be a basis for stopping rules because of the trials' value in understanding mechanisms of post-discharge worsening and antibiotic resistance. Assuming the DSMC decides to continue the trial after the interim analysis, an alpha of 0.045 will be used as the statistical significance boundary at the final analysis.

Statistical Power

To compare rates of re-hospitalization and mortality in the 6 months following hospital discharge among Kenyan children receiving 5-day azithromycin vs. placebo. The total sample size required was calculated for the primary endpoint of time to death or hospital re-admission within the 6 month post-discharge period, assuming an alpha level of 0.05, power of 0.80, and a ratio of treatment to placebo random assignment of 1:1. In SSA, it is estimated that 2-15% of children aged less than 5 years died within 6 months of hospital discharge and 15.5% of children who survived discharge from the district hospital were re-admitted with the same diagnosis within 6-months.[1 3 8] Assuming that an additional 5-10% of children are re-admitted for other conditions, we expect that re-hospitalizations will occur in 20.5 to 30.5% of children enrolled in the study. Combined with our expected fatality rate (2-15%), we expect the cumulative incidence of the combined endpoint to range from 22.5 to 45.5%.[8] Based on a previous trial of mass drug administration of a single dose of azithromycin in which a single dose of the antibiotic was associated with a 49% reduction in risk of death, we calculated sample sizes using estimates of reduction in risk ranging from 30-50% with the cumulative incidence range of 22.5 to 45.5% in the placebo-treated group, and found the sample size required ranged from 90 to 550 children per treatment arm.[15] Using the most conservative estimates of a hazard ratio of 0.70 and 22.5%

prevalence of re-admission/death, we need to enroll 1100 children in the study (550 per arm) to achieve adequate power. We will recruit an additional 300 children (≈20%) to account for possible loss to follow-up, resulting in a total planned enrollment of 1400 children, or 700 per treatment group. When considering mortality alone, and estimated mortality ranges of 2-17% among place-treated children, we will have >80% power to detect hazard ratios ≤0.5 for mortality rates of ≥ 8% and hazard ratios ≤0.6 for mortality rates ≥11% (Figure 1).

To evaluate possible mechanism(s) by which azithromycin may affect morbidity and mortality, by comparing reasons for re-hospitalization and prevalence of pathogen carriage between the randomization arms. We calculated the minimum detectable association between treatment arm and cause-specific re-hospitalizations (hospitalization due to diarrhea, acute respiratory infection, malnutrition, or malaria vs. any other) among enrolled children, assuming an alpha level of 0.05, power of 0.80, and a ratio of treatment to placebo of 1:1. Based on data from Kenya, re-hospitalization rates due to specific causes ranged from approximately 0.5% to 5.7% in the 6 month post-discharge period.[3] By not conditioning on the child having the same diagnosis as the initial hospitalization, we expect the cumulative incidence of cause-specific re-hospitalizations to range from 2.5% to 10%. With this range of outcome rates, we will be able to detect hazard ratios of 0.48 to 0.70 for the effect of azithromycin on specific severe morbidities.

We expect 56% of children in the placebo group to have *S. pneumoniae* isolated from nasopharyngeal swabs, providing ≥80% power to detect a prevalence ratio of 0.85 (or 1.15) between the two treatment arms at each time point.[28-30] Based on prevalences of *Shigella*, *Salmonella*, *Campylobacter, Cryptosporidium, Giardia* among asymptomatic children in Western Kenya, we expect 10% of children in the placebo group to have a bacterial pathogen isolated at each time point, resulting in ≥80% power to detect differences in enteric pathogen prevalences of 0.67 (1.49) at each time point.[31]

To determine whether empiric administration of azithromycin at hospital discharge increases risk of antibiotic resistance in commensal E. coli and S. pneumoniae isolates from treated children and their household contacts. We will select a random selection of 400 *E. coli* and 400 *S. pneumoniae* isolates (200 per arm) for β-lactam and macrolide resistance testing at each timepoint. We will also store all *S. pneumoniae*, *E. coli* isolates and other isolated bacteria from stool for potential future testing in the event that resistance prevalence is lower than expected. As shown in Table 4, we will have > 80% power to detect prevalence ratios > 1.1, with an ability to detect the smallest effect sizes when the prevalence of resistance in the placebo group is highest. We will enroll 300 adults in the Contact Cohort for *E. coli* and *S. pneumoniae* isolation. We expect *E.coli* to be isolated from all adults and *S.pnuemoniae* isolated from between 5-55%.[28 32 33] Assuming an alpha of .05, a 1:1 ratio of testable isolates, and a prevalence of resistance of 50% in the placebo arm, we will have 80% power to detect a 1.4-fold higher prevalence to 1.9-fold higher resistance prevalence in the contacts of azithromycin-treated children.

Table 4. Power (%) to detect prevalence ratios of macrolide and β-lactamase resistance in 200 *E.coli* and 200 *S.pneumoniae* isolates per treatment group

		**Resistance prevalence (%) in placebo group**						
**Resistance prevalence (%) in azithromycin group**		**10**	**20**	**30**	**40**	**50**	**60**	**70**
	**10**							
	**20**	80						
	**30**	>99	64					
	**40**	>99	99	55				
	**50**	>99	>99	98	48			
	**60**	>99	>99	>99	>99	52		
	**70**	>99	>99	>99	>99	98	55	
	**80**	>99	>99	>99	>99	>99	99	64

To identify correlates and intermediate markers of post-discharge mortality and hospital-readmission among hospitalized Kenyan children. Conservatively estimating a 20% loss-to-follow-up rate in the RCT and a cumulative incidence of death or re-hospitalization of 22.5%, we will have >80% power to detect hazard ratios ≥1.3 between correlates and the outcome with exposure prevalences of ≥20% or more and hazard ratios ≥1.5 for exposure prevalences <20%.

Study timeline

The trial began on June 28, 2016 and participant recruitment and follow-up will continue over a 36-month period, with anticipated final follow-up visit(s) occurring in June 2019. Primary analyses will be complete by February 2020.

Potential Challenges and Limitations

In order to ensure adequate power to detect a discernable clinically relevant difference between study groups in the primary outcome, we have combined hospital readmission with death. Preliminary studies

For peer review only - http://bmjopen.bmj.com/site/about/guidelines.xhtml

suggest that sufficient numbers of children will reach this combined outcome. However, we have incorporated an interim analysis by the DSMB to review the accrued data and an adapted sample size could be considered if the combined event frequency is less than predicted. It is possible that since most children receive antibiotics during hospitalization, the benefit anticipated with the use of azithromycin based on previous trials of mass drug administration will not be observed. However, most hospitalized children are treated with penicillins, cephalosporins, gentamicin, or cotrimoxazole while in hospital and the broad spectrum of activity (including malaria prevention) and long half-life of azithromycin suggest that there may be additive treatment and/or prophylactic benefit. Similarly, children may receive azithromycin during follow up - either as treatment for an illness or because the caregiver sought out azithromycin upon learning of the hypothesis – and this azithromycin use may lead to contamination in the placebo-arm. After discharge, it is difficult to ensure adherence with the full 5-day treatment course. We will measure adherence using three different measures (text message responses, bottle check boxes, and caregiver-report at follow-up visits) although all are limited by caregiver-report. In addition, the mortality benefit of azithromycin observed in Ethiopia was from a single dose and in this study the first dose will be directly observed.[15] While relying on caregiver report of mortality and morbidity may lead to bias due to outcome misclassification, this misclassification should not differ between randomization arms and therefore will be non-differential. Further hospital records will be used when available to determine diagnoses. Finally, resistance prevalence may be lower than predicted, limiting power to detect clinically relevant differences in resistance prevalence between the intervention arms. We will store all isolates in the event that a greater number of isolates are needed for antibiotic resistance testing.

Regulatory Authorities

This study has received IRB approval by the University of Washington Human Subjects Division (HSD), KEMRI Scientific and Ethics Review Unit (SERU), and the Kenya Pharmacy and Poisons Board. The clinical trial is also registered with clinicaltrials.gov (NCT02414399). Any modifications to the study protocol or consent materials will be submitted for approval all regulatory authorities before implementation. Westat® will provide external clinical, pharmacy, and laboratory monitoring.

Dissemination

Results of this study will be disseminated by publication in a peer-reviewed scientific journal, presented at relevant academic conferences, and amongst participating partners and health facilities in Kenya.

Author's contributions

JLW, PBP, GJS, BAR, BOS, and RN conceived of this trial and developed of this study protocol. JLW and BOS are study co-Principal Investigators and PBP is the Project Director; BAR oversaw the statistical analyses plans; JBB developed the CEA plan; KDT developed procedures for ascertaining and reporting SAEs; CJM developed procedures related to blood specimen procedures and drug adherence measurement. GJS, CJM, RN, and PBP provide scientific expertise. RLB and MA are involved in collection and management of the data. MA and PBP coordinate and oversee implementation of all clinical study procedures and SK, with assistance from DR, oversees all laboratory procedures. All authors contributed to the development of this manuscript and/or study procedures, and to reading and approving the final version for publication.

Funding statement

This work was funded by the National Institutes of Health Eunice Kennedy Shriver National Institute of Child Health & Human Development, grant number R01 HD079695.

Acknowledgements

Pfizer donated the Zithromax® to be used in this clinical trial, and Copan Diagnostics donated all rectal swabs and Cary-Blair media. Investigators from KEMRI-Wellcome Trust Kilifi, Jay Berkley, Anthony Scott, Joseph Waichungo, Angela Karani, Donald Akech, and Horace Gumba provided microbiology expertise and training in nasopharyngeal swab collection, STGG media preparation, and laboratory quality assurance and control. Liru Meshack Wekesa and George Bogonko provide clinical expertise and facilitate the study's integration into pediatric wards at the two hospitals. Alex Awuor and Caleb Okonji, with the support of Richard Omore, provided training in anthropometric measurement. We are extremely thankful to Dr. Philip Walson who developed azithromycin dosing regimens. Hannah Atlas and Stephanie Belanger contributed to the standard

For peer review only

operating procedure and case report form development and implementation. Gillian Levine played an invaluable role in the proposal development.

Competing interests statement

None of the authors or study co-investigators have any competing interests to declare.

Figure Legend

Figure 1. Power and detectable hazard ratios assuming a range of mortality rates from 2-17%

REFERENCES

1. Moisi JC, Gatakaa H, Berkley JA, et al. Excess child mortality after discharge from hospital in Kilifi, Kenya: a retrospective cohort analysis. *Bull World Health Organ* 2011;89(10):725-32, 32A. doi: 10.2471/BLT.11.089235
2. Veirum JE, Sodeman M, Biai S, et al. Increased mortality in the year following discharge from a paediatric ward in Bissau, Guinea-Bissau. *Acta Paediatr* 2007;96(12):1832-8. doi: 10.1111/j.1651-2227.2007.00562.x [published Online First: 2007/11/16]
3. Snow RW, Howard SC, Mung'Ala-Odera V, et al. Paediatric survival and re-admission risks following hospitalization on the Kenyan coast. *Trop Med Int Health* 2000;5(5):377-83. [published Online First: 2000/07/08]
4. Zucker JR, Lackritz EM, Ruebush TK, 2nd, et al. Childhood mortality during and after hospitalization in western Kenya: effect of malaria treatment regimens. *Am J Trop Med Hyg* 1996;55(6):655-60. [published Online First: 1996/12/01]
5. Islam MA, Rahman MM, Mahalanabis D, et al. Death in a diarrhoeal cohort of infants and young children soon after discharge from hospital: risk factors and causes by verbal autopsy. *J Trop Pediatr* 1996;42(6):342-7. [published Online First: 1996/12/01]
6. Villamor E, Misegades L, Fataki MR, et al. Child mortality in relation to HIV infection, nutritional status, and socio-economic background. *Int J Epidemiol* 2005;34(1):61-8. doi: 10.1093/ije/dyh378 [published Online First: 2005/01/15]
7. Phiri KS, Calis JC, Faragher B, et al. Long term outcome of severe anaemia in Malawian children. *PLoS One* 2008;3(8):e2903. doi: 10.1371/journal.pone.0002903 [published Online First: 2008/08/07]
8. Wiens MO, Pawluk S, Kissoon N, et al. Pediatric post-discharge mortality in resource poor countries: a systematic review. *PLoS One* 2013;8(6):e66698. doi: 10.1371/journal.pone.0066698 [published Online First: 2013/07/05]
9. Bwakura-Dangarembizi M, Kendall L, Bakeera-Kitaka S, et al. A randomized trial of prolonged co-trimoxazole in HIV-infected children in Africa. *The New England journal of medicine* 2014;370(1):41-53. doi: 10.1056/NEJMoa1214901
10. Walker AS, Mulenga V, Ford D, et al. The impact of daily cotrimoxazole prophylaxis and antiretroviral therapy on mortality and hospital admissions in HIV-infected Zambian children. *Clin Infect Dis* 2007;44(10):1361-7. doi: 10.1086/515396 [published Online First: 2007/04/20]
11. Chintu C, Bhat GJ, Walker AS, et al. Co-trimoxazole as prophylaxis against opportunistic infections in HIV-infected Zambian children (CHAP): a double-blind randomised placebo-controlled trial. *Lancet* 2004;364(9448):1865-71. doi: 10.1016/S0140-6736(04)17442-4 [published Online First: 2004/11/24]
12. Trehan I, Goldbach HS, LaGrone LN, et al. Antibiotics as part of the management of severe acute malnutrition. *The New England journal of medicine* 2013;368(5):425-35. doi: 10.1056/NEJMoa1202851
13. Lockman S, Hughes M, Powis K, et al. Effect of co-trimoxazole on mortality in HIV-exposed but uninfected children in Botswana (the Mpepu Study): a double-blind, randomised, placebo-controlled trial. *Lancet Glob Health* 2017;5(5):e491-e500. doi: 10.1016/s2214-109x(17)30143-2 [published Online First: 2017/04/12]
14. Berkley JA, Ngari M, Thitiri J, et al. Daily co-trimoxazole prophylaxis to prevent mortality in children with complicated severe acute malnutrition: a multicentre, double-blind, randomised placebo-controlled trial. *Lancet Glob Health* 2016;4(7):e464-73. doi: 10.1016/S2214-109X(16)30096-1

For peer review only

15. Porco TC, Gebre T, Ayele B, et al. Effect of mass distribution of azithromycin for trachoma control on overall mortality in Ethiopian children: a randomized trial. *JAMA* 2009;302(9):962-8. doi: 10.1001/jama.2009.1266 [published Online First: 2009/09/03]
16. Keenan JD, Ayele B, Gebre T, et al. Childhood mortality in a cohort treated with mass azithromycin for trachoma. *Clin Infect Dis* 2011;52(7):883-8. doi: 10.1093/cid/cir069 [published Online First: 2011/03/24]
17. O'Brien KL, Nohynek H, World Health Organization Pneumococcal Vaccine Trials Carraige Working G. Report from a WHO working group: standard method for detecting upper respiratory carriage of Streptococcus pneumoniae. *Pediatr Infect Dis J* 2003;22(2):133-40. doi: 10.1097/01.inf.0000048676.93549.d1
18. Satzke C, Turner P, Virolainen-Julkunen A, et al. Standard method for detecting upper respiratory carriage of Streptococcus pneumoniae: updated recommendations from the World Health Organization Pneumococcal Carriage Working Group. *Vaccine* 2013;32(1):165-79. doi: 10.1016/j.vaccine.2013.08.062
19. Serina P, Riley I, Stewart A, et al. A shortened verbal autopsy instrument for use in routine mortality surveillance systems. *BMC Med* 2015;13:302. doi: 10.1186/s12916-015-0528-8
20. Serina P, Riley I, Stewart A, et al. Improving performance of the Tariff Method for assigning causes of death to verbal autopsies. *BMC Med* 2015;13:291. doi: 10.1186/s12916-015-0527-9
21. WHO. Making choices in health: WHO guide to cost-effectiveness analysis. 2003.
22. Murray CJ, Vos T, Lozano R, et al. Disability-adjusted life years (DALYs) for 291 diseases and injuries in 21 regions, 1990-2010: a systematic analysis for the Global Burden of Disease Study 2010. *Lancet* 2012;380(9859):2197-223. doi: 10.1016/s0140-6736(12)61689-4 [published Online First: 2012/12/19]
23. Salomon JA, Vos T, Hogan DR, et al. Common values in assessing health outcomes from disease and injury: disability weights measurement study for the Global Burden of Disease Study 2010. *Lancet* 2012;380(9859):2129-43. doi: 10.1016/s0140-6736(12)61680-8 [published Online First: 2012/12/19]
24. Gold M, Siegel JE, Russell LB, Weinstein MC. Cost-effectiveness in health and medicine. Oxford University Press: New York 1996.
25. Drummond MF, Torrance GW, O'Brien BJ, Stoddart GL. Methods for the economic evaluation of healthcare programmes, 3rd edition. Oxford University Press: New York 2005.
26. Laxminarayan R, Mills AJ, Breman JG, et al. Advancement of global health: key messages from the Disease Control Priorities Project. *Lancet* 2006;367(9517):1193-208. doi: 10.1016/s0140-6736(06)68440-7 [published Online First: 2006/04/18]
27. Jamison DT, Measham A, Alleyne G, Claeson M, et al. Disease Control Priorities in Developing Countries, 2nd edition. Oxford University Press, 2006.
28. Abdullahi O, Karani A, Tigoi CC, et al. The prevalence and risk factors for pneumococcal colonization of the nasopharynx among children in Kilifi District, Kenya. *PLoS One* 2012;7(2):e30787. doi: 10.1371/journal.pone.0030787 [published Online First: 2012/03/01]
29. Rodrigues F, Foster D, Caramelo F, et al. Progressive changes in pneumococcal carriage in children attending daycare in Portugal after 6 years of gradual conjugate vaccine introduction show falls in most residual vaccine serotypes but no net replacement or trends in diversity. *Vaccine* 2012;30(26):3951-6. doi: 10.1016/j.vaccine.2012.03.058 [published Online First: 2012/04/07]
30. Grivea IN, Tsantouli AG, Michoula AN, et al. Dynamics of Streptococcus pneumoniae nasopharyngeal carriage with high heptavalent pneumococcal conjugate vaccine coverage in Central Greece. *Vaccine* 2011;29(48):8882-7. doi: 10.1016/j.vaccine.2011.09.074 [published Online First: 2011/10/04]
31. Skalet AH, Cevallos V, Ayele B, et al. Antibiotic selection pressure and macrolide resistance in nasopharyngeal Streptococcus pneumoniae: a cluster-randomized clinical trial. *PLoS Med* 2010;7(12):e1000377. doi: 10.1371/journal.pmed.1000377 [published Online First: 2010/12/24]
32. Adetifa IM, Antonio M, Okoromah CA, et al. Pre-vaccination nasopharyngeal pneumococcal carriage in a Nigerian population: epidemiology and population biology. *PLoS One* 2012;7(1):e30548. doi: 10.1371/journal.pone.0030548 [published Online First: 2012/02/01]
33. Hill PC, Akisanya A, Sankareh K, et al. Nasopharyngeal carriage of Streptococcus pneumoniae in Gambian villagers. *Clin Infect Dis* 2006;43(6):673-9. doi: 10.1086/506941 [published Online First: 2006/08/17]

For peer review only

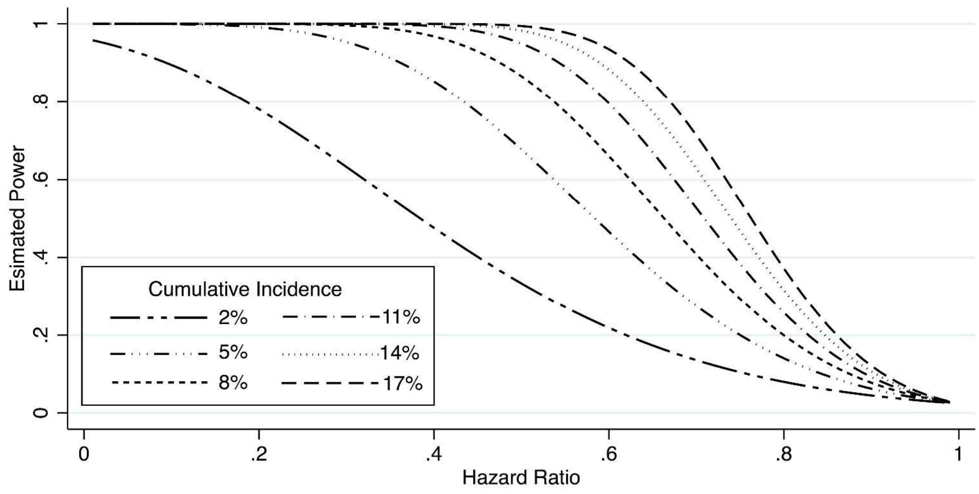

Figure 1. Power and detectable hazard ratios assuming a range of mortality rates from 2-17%

644x332mm (300 x 300 DPI)

For peer review only

STANDARD PROTOCOL ITEMS: RECOMMENDATIONS FOR INTERVENTIONAL TRIALS

SPIRIT 2013 Checklist: Recommended items to address in a clinical trial protocol and related documents*

Section/item	Item No	Description	Addressed on page number
**Administrative information**			
Title	1	Descriptive title identifying the study design, population, interventions, and, if applicable, trial acronym	______________
Trial registration	2a	Trial identifier and registry name. If not yet registered, name of intended registry	______________
	2b	All items from the World Health Organization Trial Registration Data Set	______________
Protocol version	3	Date and version identifier	______________
Funding	4	Sources and types of financial, material, and other support	______________
Roles and responsibilities	5a	Names, affiliations, and roles of protocol contributors	______________
	5b	Name and contact information for the trial sponsor	______________
	5c	Role of study sponsor and funders, if any, in study design; collection, management, analysis, and interpretation of data; writing of the report; and the decision to submit the report for publication, including whether they will have ultimate authority over any of these activities	______________
	5d	Composition, roles, and responsibilities of the coordinating centre, steering committee, endpoint adjudication committee, data management team, and other individuals or groups overseeing the trial, if applicable (see Item 21a for data monitoring committee)	______________

For peer review only

Introduction

Background and rationale	6a	Description of research question and justification for undertaking the trial, including summary of relevant studies (published and unpublished) examining benefits and harms for each intervention	______________
	6b	Explanation for choice of comparators	______________
Objectives	7	Specific objectives or hypotheses	______________
Trial design	8	Description of trial design including type of trial (eg, parallel group, crossover, factorial, single group), allocation ratio, and framework (eg, superiority, equivalence, noninferiority, exploratory)	______________

Methods: Participants, interventions, and outcomes

Study setting	9	Description of study settings (eg, community clinic, academic hospital) and list of countries where data will be collected. Reference to where list of study sites can be obtained	______________
Eligibility criteria	10	Inclusion and exclusion criteria for participants. If applicable, eligibility criteria for study centres and individuals who will perform the interventions (eg, surgeons, psychotherapists)	______________
Interventions	11a	Interventions for each group with sufficient detail to allow replication, including how and when they will be administered	______________
	11b	Criteria for discontinuing or modifying allocated interventions for a given trial participant (eg, drug dose change in response to harms, participant request, or improving/worsening disease)	______________
	11c	Strategies to improve adherence to intervention protocols, and any procedures for monitoring adherence (eg, drug tablet return, laboratory tests)	______________
	11d	Relevant concomitant care and interventions that are permitted or prohibited during the trial	______________
Outcomes	12	Primary, secondary, and other outcomes, including the specific measurement variable (eg, systolic blood pressure), analysis metric (eg, change from baseline, final value, time to event), method of aggregation (eg, median, proportion), and time point for each outcome. Explanation of the clinical relevance of chosen efficacy and harm outcomes is strongly recommended	______________
Participant timeline	13	Time schedule of enrolment, interventions (including any run-ins and washouts), assessments, and visits for participants. A schematic diagram is highly recommended (see Figure)	______________

For peer review only - http://bmjopen.bmj.com/site/about/guidelines.xhtml

Sample size	14	Estimated number of participants needed to achieve study objectives and how it was determined, including clinical and statistical assumptions supporting any sample size calculations	______________
Recruitment	15	Strategies for achieving adequate participant enrolment to reach target sample size	______________

Methods: Assignment of interventions (for controlled trials)

Allocation:			
Sequence generation	16a	Method of generating the allocation sequence (eg, computer-generated random numbers), and list of any factors for stratification. To reduce predictability of a random sequence, details of any planned restriction (eg, blocking) should be provided in a separate document that is unavailable to those who enrol participants or assign interventions	______________
Allocation concealment mechanism	16b	Mechanism of implementing the allocation sequence (eg, central telephone; sequentially numbered, opaque, sealed envelopes), describing any steps to conceal the sequence until interventions are assigned	______________
Implementation	16c	Who will generate the allocation sequence, who will enrol participants, and who will assign participants to interventions	______________
Blinding (masking)	17a	Who will be blinded after assignment to interventions (eg, trial participants, care providers, outcome assessors, data analysts), and how	______________
	17b	If blinded, circumstances under which unblinding is permissible, and procedure for revealing a participant's allocated intervention during the trial	______________

Methods: Data collection, management, and analysis

Data collection methods	18a	Plans for assessment and collection of outcome, baseline, and other trial data, including any related processes to promote data quality (eg, duplicate measurements, training of assessors) and a description of study instruments (eg, questionnaires, laboratory tests) along with their reliability and validity, if known. Reference to where data collection forms can be found, if not in the protocol	______________
	18b	Plans to promote participant retention and complete follow-up, including list of any outcome data to be collected for participants who discontinue or deviate from intervention protocols	______________

Data management	19	Plans for data entry, coding, security, and storage, including any related processes to promote data quality (eg, double data entry; range checks for data values). Reference to where details of data management procedures can be found, if not in the protocol	______________
Statistical methods	20a	Statistical methods for analysing primary and secondary outcomes. Reference to where other details of the statistical analysis plan can be found, if not in the protocol	______________
	20b	Methods for any additional analyses (eg, subgroup and adjusted analyses)	______________
	20c	Definition of analysis population relating to protocol non-adherence (eg, as randomised analysis), and any statistical methods to handle missing data (eg, multiple imputation)	______________
**Methods: Monitoring**			
Data monitoring	21a	Composition of data monitoring committee (DMC); summary of its role and reporting structure; statement of whether it is independent from the sponsor and competing interests; and reference to where further details about its charter can be found, if not in the protocol. Alternatively, an explanation of why a DMC is not needed	______________
	21b	Description of any interim analyses and stopping guidelines, including who will have access to these interim results and make the final decision to terminate the trial	______________
Harms	22	Plans for collecting, assessing, reporting, and managing solicited and spontaneously reported adverse events and other unintended effects of trial interventions or trial conduct	______________
Auditing	23	Frequency and procedures for auditing trial conduct, if any, and whether the process will be independent from investigators and the sponsor	______________
**Ethics and dissemination**			
Research ethics approval	24	Plans for seeking research ethics committee/institutional review board (REC/IRB) approval	______________
Protocol amendments	25	Plans for communicating important protocol modifications (eg, changes to eligibility criteria, outcomes, analyses) to relevant parties (eg, investigators, REC/IRBs, trial participants, trial registries, journals, regulators)	______________

For peer review only - http://bmjopen.bmj.com/site/about/guidelines.xhtml

Consent or assent	26a	Who will obtain informed consent or assent from potential trial participants or authorised surrogates, and how (see Item 32)	________
	26b	Additional consent provisions for collection and use of participant data and biological specimens in ancillary studies, if applicable	________
Confidentiality	27	How personal information about potential and enrolled participants will be collected, shared, and maintained in order to protect confidentiality before, during, and after the trial	________
Declaration of interests	28	Financial and other competing interests for principal investigators for the overall trial and each study site	________
Access to data	29	Statement of who will have access to the final trial dataset, and disclosure of contractual agreements that limit such access for investigators	________
Ancillary and post-trial care	30	Provisions, if any, for ancillary and post-trial care, and for compensation to those who suffer harm from trial participation	________
Dissemination policy	31a	Plans for investigators and sponsor to communicate trial results to participants, healthcare professionals, the public, and other relevant groups (eg, via publication, reporting in results databases, or other data sharing arrangements), including any publication restrictions	________
	31b	Authorship eligibility guidelines and any intended use of professional writers	________
	31c	Plans, if any, for granting public access to the full protocol, participant-level dataset, and statistical code	________
**Appendices**			
Informed consent materials	32	Model consent form and other related documentation given to participants and authorised surrogates	________
Biological specimens	33	Plans for collection, laboratory evaluation, and storage of biological specimens for genetic or molecular analysis in the current trial and for future use in ancillary studies, if applicable	________

*It is strongly recommended that this checklist be read in conjunction with the SPIRIT 2013 Explanation & Elaboration for important clarification on the items. Amendments to the protocol should be tracked and dated. The SPIRIT checklist is copyrighted by the SPIRIT Group under the Creative Commons "Attribution-NonCommercial-NoDerivs 3.0 Unported" license.

For peer review only - http://bmjopen.bmj.com/site/about/guidelines.xhtml

BMJ Open

Azithromycin to prevent post-discharge morbidity and mortality in Kenyan children: A protocol for a randomized, double-blind, placebo-controlled trial (the Toto Bora trial)

Journal:	*BMJ Open*
Manuscript ID	bmjopen-2017-019170.R2
Article Type:	Protocol
Date Submitted by the Author:	08-Nov-2017
Complete List of Authors:	Pavlinac, Patricia; University of Washington, Global Health<br>Singa, Benson; Kenya Medical Research Institute; Childhood Acute Illness and Nutrition Network<br>John-Stewart, G; University of Washington, Global Health, Epidemiology, Pediatrics, Allergy and Infectious Disease<br>Richardson, BA; University of Washington, Global Health, BIostatistics<br>Brander, Rebecca; University of Washington, Epidemiology<br>McGrath, Christine; University of Washington, Global Health<br>Tickell, Kirkby; University of Washington, Global Health; Childhood Acute Illness and Nutrition Network<br>Amondi, Mary; Kenya Medical Research Institute<br>Rwigi, Doreen; Kenya Medical Research Institute<br>Babigumira, Joseph; University of Washington, Global Health, Pharmacy<br>Kariuki, Sam; Kenya Medical Research Institute<br>Nduati, Ruth; University of Nairobi<br>Walson, Judd; University of Washington, Department of Global Health
**Primary Subject Heading**:	Global health
Secondary Subject Heading:	Paediatrics, Infectious diseases
Keywords:	Child mortality, Post-discharge interventions, Toto Bora Trial, Targeted empiric antibiotic therapy

SCHOLARONE™
Manuscripts

Title: Azithromycin to prevent post-discharge morbidity and mortality in Kenyan children: A protocol for a randomized, double-blind, placebo-controlled trial (the Toto Bora trial)

Running head: AZM to prevent post-discharge morbidity and mortality

Authors: Pavlinac PB[1], Singa BO[7,8], John-Stewart GC[1-4], Richardson BA[1,5], Brander RL[2], McGrath CJ[1], Tickell KD[1,8], Amondi M[7], Rwigi D[7], Babigumira JB[1,6], Kariuki S[7], Nduati R[9], Walson JL[1-4,8]

Affiliations: Departments of [1]Global Health, [2]Epidemiology, [3]Pediatrics, [4]Allergy and Infectious Disease, [5]Biostatistics, [6]Pharmacy, University of Washington, Seattle, WA, USA; [7]Kenya Medical Research Institute, Nairobi, Kenya; [8]The Childhood Acute Illness & Nutrition Network, Nairobi, Kenya; [9] University of Nairobi, Nairobi, Kenya

Correspondence: Patricia Pavlinac, 325 9th Ave, Box 359931, Seattle, WA 98104 (ppav@uw.edu)

Sponsored by: This trial is sponsored by the National Institutes of Health Eunice Kennedy Shriver National Institute of Child Health & Human Development (R01 HD079695) and drug donation by Pfizer IIR #WI201906. JLW is supported by the Childhood Acute Illness and Nutrition Network (CHAIN, OPP1131320). GJS is supported by a National Institute of Health mentoring award (grant number K24-HD054314) and PBP and GJS are supported by the International Core of the University of Washington Center for AIDS Research (CFAR; Seattle, WA, USA), an NIH funded program (P30 AI027757).

Word count: 5,730 (excluding tables and figures)

For peer review only

ABSTRACT

Introduction: Child mortality due to infectious diseases remains unacceptably high in much of sub-Saharan Africa. Children who are hospitalized represent an accessible population at particularly high-risk of death, both during and following hospitalization. Hospital discharge may be a critical time point at which targeted use of antibiotics could reduce morbidity and mortality in high-risk children.

Methods and analysis: In this randomized, double-blind, placebo-controlled trial (Toto Bora Trial), 1400 children aged 1 to 59 months discharged from hospitals in western Kenya, in Kisii and Homa Bay, will be randomized to either a 5-day course of azithromycin or placebo to determine whether a short-course of azithromycin reduces rates of re-hospitalization and/or death in the subsequent 6-month period. The primary analysis will be modified intention-to-treat and will compare the rates of re-hospitalization or death in children treated with azithromycin or placebo using Cox proportional hazard regression. The trial will also evaluate the effect of a short course of azithromycin on enteric and nasopharyngeal infections and cause-specific morbidities. We will also identify risk factors for post-discharge morbidity and mortality and subpopulations most likely to benefit from post-discharge antibiotic use. Antibiotic resistance in *Escherichia coli* and *Streptococcus pneumoniae* among enrolled children and their primary caregivers will also be assessed and cost-effectiveness analyses performed to inform policy decisions.

Ethics and dissemination: Study procedures were reviewed and approved by the institutional review boards of the Kenya Medical Research Institute, the University of Washington, and the Kenyan Pharmacy and Poisons Board. The study is being externally monitored and a data safety and monitoring committee has been assembled to monitor patient safety and to evaluate the efficacy of the intervention. The results of this trial will be published in peer-reviewed scientific journals and presented at relevant academic conferences and to key stakeholders.

Trial registration number: NCT02414399

Key words: child mortality, Toto Bora, targeted empiric antibiotic therapy, post-discharge interventions

STRENGTHS AND LIMITATIONS OF THIS STUDY

Strengths

- Randomized, placebo-controlled, double-blinded design and modified intention-to-treat analysis will ensure unbiased treatment effect measure
- Comprehensive data are collected, including biological specimens for all child participants and a subset of adult caregivers, for analyses of mechanisms of post-discharge morbidity and mortality, subsets of children most likely to benefit from the antibiotic, as well as assessments of antibiotic resistance and cost-effectiveness
- Results will likely be generalizable due to the limited exclusion criteria, large sample size, and multiple study sites

Limitations

- Causes of death and re-hospitalization may be misclassified due to limited availability of medical records and recall bias in caregiver report
- The primary endpoint of this study is a combined outcome of re-hospitalization and death, which, while improving statistical power, may present challenges for interpretation
- Children in both intervention arms may receive other antibiotics over the course of follow-up

For peer review only

BACKGROUND

Close to 3 million deaths occur annually in children less than 5 years of age in sub-Saharan Africa (SSA), over half of which are attributed to infectious causes.[1] Children who were recently hospitalized have mortality rates 6 to 8-fold higher than similarly-aged children from the same community.[2-4] Post-discharge mortality rates as high as 15% have been documented in the 12 months following discharge, with mortality risk remaining elevated up to two years post-discharge.[5-9] Children who are very young, malnourished, or HIV-infected are at particularly high risk of post-discharge mortality within the 3 months following discharge.[2-5 7-9] Children being discharged from hospital in SSA may represent an accessible high-risk population in which to target interventions to reduce mortality and morbidity.

Targeted antibiotic interventions, including the use of cotrimoxazole among HIV-infected children and the use of amoxicillin or cefdinir among children with severe acute malnutrition (SAM), have been shown to reduce morbidity and mortality in these specific vulnerable populations.[10-13] Other trials of targeted antibiotic use in vulnerable populations, including cotrimoxazole in HIV-exposed uninfected (HEU) children and in children with SAM, have failed to demonstrate a mortality benefit.[14 15] In contrast, non-targeted mass drug administration of a single dose of azithromycin halved mortality rates among Ethiopian children living in communities randomized to receive the antibiotic.[16 17] Concerns about the potential emergence of antibiotic resistance, possible toxicity, and feasibility of delivery are barriers to community-wide antibiotic distribution strategies.

A short-course of azithromycin given to children with recent severe illness being discharged from hospital may optimize benefit while reducing both individual and population level risks. Azithromycin may reduce post-discharge morbidity and mortality through infection related mechanisms such as treating undiagnosed, incompletely treated or nosocomial infections or by protecting against new or recrudescent infections that occur during recovery. Azithromycin may also act through non-antimicrobial pathways such as by anti-inflammatory and/or immune-modulatory effects.

OBJECTIVE

The primary objective of this double-blind, placebo-controlled, randomized controlled trial (RCT) is to determine whether a 5-day course of azithromycin in children age 1 to 59 months discharged from hospital in western Kenya reduces rates of re-hospitalizations and/or death in the subsequent six months. The secondary objectives are (1) to evaluate possible mechanism(s) by which azithromycin may affect morbidity and mortality, by comparing reasons for re-hospitalization and prevalence of enteric and nasopharyngeal infections between the randomization arms; (2) to determine whether empiric administration of azithromycin at hospital discharge increases risk of antibiotic resistance in commensal *Escherichia coli* (*E. coli*) and *Streptococcus pneumoniae (S. pneumoniae)* isolates from treated children and their primary caregiver; (3) to identify correlates and intermediate markers of post-discharge mortality and hospital readmission; (4) to determine the cost-effectiveness of post-discharge administration of a 5-day course of azithromycin in settings of varying antibiotic use, re-hospitalization rates, and mortality rates; and (5) to create a repository of stool, nasopharyngeal, and blood specimens from highly characterized, recently discharged children, half of whom are treated with azithromycin, to be used to address future research questions.

METHODS

Reporting of this study protocol has been verified in accordance with the SPIRIT (Standard Protocol Items for Randomized Trials) recommendations.

Eligibility

Children age 1 to 59 months old weighing at least 2 kg who have been hospitalized, and subsequently discharged, will be eligible for inclusion. Caregivers of potentially eligible children must be at least 18 years of age or classified as an emancipated minor and be willing to participate in the Contact Cohort if randomly selected. Children will be excluded if: azithromycin is contraindicated (children taking or prescribed other macrolide antibiotics, such as erythromycin or clarithromycin, or the protease inhibitor, lopinavir); they were admitted to hospital for a trauma, injury, or a birth defect; they do not plan to remain in the study site catchment area for at least 6 months; the legal guardian does not provide consent; or if a sibling was enrolled in the trial on the same day of discharge.

For peer review only

Recruitment

Children will be recruited from the inpatient wards of health facilities in Kisii and Homa Bay Counties where study staff will accompany hospital staff on ward rounds to identify children being discharged each day. All discharged children, as determined by the onsite hospital clinicians, will be screened by study staff during working hours. If the caregiver is interested in participating and indicates consent for screening, the study staff will screen the child for eligibility, and if eligible, will obtain informed consent for study participation. Informed consent includes an explanation of the potential risks and benefits of the study and additional provision for use of participant data and samples for future studies, and will be conducted in the language of the respondent's choosing (English, Kiswahili, Kisii, or Luo). The parent or guardian (primary caregiver) must sign written informed consent (or provide a witnessed thumbprint if not literate) prior to enrollment.

Enrollment

Children will be enrolled at the time of discharge by the clinical staff. At enrollment, primary caregivers will be interviewed to assess demographic information, medical history, and detailed contact information for the child (Table 1). Medical records will also be used to abstract information from the hospitalization (including presenting diagnosis, medical management, length of stay, procedures performed, relevant medical history, physical examination, and laboratory data). All enrolled participants will undergo a physical examination performed by the study clinician, including measurement of height (in children ≥ 24 months), length (in children <24 months), weight, and mid-upper arm circumference (MUAC), each of which will be measured three times. HIV status will be obtained from medical records or from performed testing if records are not available. Detailed home location and contact information will be collected to enable patient tracing.

Table 1. Summary of data collected among enrolled children at each study visit

Enrollment visit (hospital discharge)	3 month follow up visit	6 month follow up visit	Unscheduled visits
• Questionnaire of sociodemographic, clinical history, treatments prescribed in hospital and at discharge, hospitalization costs, dietary factors, household factors, and environmental exposures<br>• Physical exam<br>• Anthropometry<br>• Abstraction of medical records (if re-hospitalized)<br>• Verbal autopsy (or abstracted medical records)<br>• Heel/finger prick (HIV and malaria)<br>• Stool collection (*Shigella*, *Salmonella*, *Campylobacter*, *Escherichia coli*, *Cryptosporidium*, and Giardia)<br>• Nasopharyngeal swab collection (*Streptococcus pneumoniae*)	• Questionnaire of study drug administration, and reported illnesses, hospitalization costs, change in clinical history, and treatments since last visit<br>• Physical exam<br>• Anthropometry<br>• Abstraction of medical records (if re-hospitalized)<br>• Verbal autopsy (or abstracted medical records)<br>• Heel/finger prick (HIV and malaria)<br>• Stool collection (*Shigella*, *Salmonella*, *Campylobacter*, *Escherichia coli*, *Cryptosporidium*, and Giardia)<br>• Nasopharyngeal swab collection (*Streptococcus pneumoniae*)	• Questionnaire of reported illnesses, hospitalization costs, change in clinical history, and treatments since last visit<br>• Physical exam<br>• Anthropometry<br>• Abstraction of medical records (if re-hospitalized)<br>• Verbal autopsy (or abstracted medical records)<br>• Heel/finger prick (HIV and malaria, sickle-cell)<br>• Stool collection (*Shigella*, *Salmonella*, *Campylobacter*, *Escherichia coli*, *Cryptosporidium*, and Giardia)<br>• Nasopharyngeal swab collection (*Streptococcus pneumoniae*)	• Questionnaire of reported illnesses since last scheduled visit, change in clinical history, and treatments since last visit<br>• Abstraction of medical records (if re-hospitalized)<br>• Verbal autopsy (or abstracted medical records)

Specimen collection

Specimens will be collected at enrollment (prior to study medication administration, as well as at 3 and 6-month follow-up visits). All children will also be asked to provide a whole stool for enteric pathogen identification and storage. Stool samples will be divided within one hour of collection for the following purposes: 1) placed in Cary-Blair for eventual bacterial culture (FecalSwab Cary-Blair Collection and Transport Systemt™, Copan Diagnostics), 2) immediately tested for *Giardia* and *Cryptosporidium* using the immunoassay (Quik Chek™, Alere) and 3) placed in -80°C storage for future molecular determination of pathogen or commensal flora and markers of gut function (whole stool for these purposes will be divided into two separate vials). If a child cannot produce whole stool, two flocked rectal swabs (Pedatric FLOQswab™,

For peer review only - http://bmjopen.bmj.com/site/about/guidelines.xhtml

Copan Diagnostics) will be collected and one placed in Cary-Blair and the other stored in -80℃ for future analyses.

One flocked dry nasopharyngeal swab (Copan Diagnostics) will also be collected from all enrolled children at each time point, immediately placed in skim milk, tryptone, glucose, and glycerine (STGG) media, and frozen (-80℃) within 1 hour of collection for future *S.pnuemoniae* culture.[18 19] Primary caregivers in the Contact Cohort will also be asked to provide a stool sample (or 2 rectal swabs) and nasopharyngeal sample at each visit for testing and storage as described above.

Venous blood (up to 1 teaspoon [5mL]) will be collected from all enrolled children at each time point into EDTA tubes and separated for the following purposes: 1) 0.5mL for immediate HIV-testing (if indicated according to Kenyan Ministry of Health guidelines), 2) 0.4mL for a thin malaria smear which will be stored at room temperature, 3) 0.4mL for a dried blood spot, and 4) 2-4mL for plasma and buffy coat isolation and -80℃ storage. Blood will also be collected from primary caregivers for HIV-testing if indicated.

Randomization

Block randomization (1:1) in random sized blocks of no more than 10, stratified by site, will be used. Primary randomization will include allocation to the Contact Cohorts at a ratio of 1:5 (resulting in 150 per treatment arm). Each subject will be assigned a Patient Identification Number (PID), and the randomization code linking each PID to the allocated treatment will be generated by a designated statistician and maintained by the University of Washington Research Pharmacy. Study participants, investigators (other than the statistician), the study staff, hospital clinicians, and persons involved in data management or analysis will remain blinded to the allocation group during all data collection phases of the study.

Intervention

Enrolled children will be prescribed a 5-day course of oral suspension formulation azithromycin (Zithromax® from Pfizer, 10 mg/kg on day 1, followed by 5mg/kg/day on days 2-5) or identically appearing and tasting placebo at discharge. Identically appearing bottles will be pre-labelled with the PID. Dosing ranges were determined such that a given child would never be under-dosed or over-dosed by more than 20% of the weight-specific intended dose (Table 2). The day 1 dose will be split in half and the first half administered by the study clinician (to be observed by the caregiver) followed by the second half administered by the caregiver under careful observation of the study staff. Day 2-5 doses will be administered by caregivers at their home. Caregivers will be provided with visual instructions in the language of their choosing (English, Kiswahili, Luo, Kisii).

Table 2. Azithromycin dosing chart by child weight

Weight (kg)	Day 1 dose (mL)	Day 2-5 dose (mL)
**2.0**	0.25 x 2	0.25
**2.1-2.4**	0.30 x 2	0.30
**2.5-2.8**	0.35 x 2	0.35
**2.9-3.2**	0.40 x 2	0.40
**3.3-3.6**	0.45 x 2	0.45
**3.7-4.0**	0.50 x 2	0.50
**4.1-4.8**	0.60 x 2	0.6
**4.9-5.6**	0.70 x 2	0.7
**5.7-6.8**	0.85 x 2	0.85
**6.9-8.0**	1.0 x 2	1.0
**8.1-9.6**	1.2 x 2	1.2
**9.7-11.2**	1.4 x 2	1.4
**11.3-13.6**	1.6 x 2	1.6
**13.7-16.0**	2.0 x 2	2.0
**16.1-19.2**	2.4 x 2	2.4
**19.3-23.2**	2.9 x 2	2.9
**23.3-25.0**	3.2 x 2	3.2

Automated daily text message drug administration reminders will be sent for the four days following discharge and caregivers asked to respond with whether or not the child took the daily dose. The response text message will be free of charge to caregivers and caregivers will be reimbursed for each response at the final study visit. Caregivers are also asked to record each administered dose on the bottle and to return bottles at the 3-month follow-up visit. The questionnaire administered during the 3-month follow up visit also includes questions about how many doses of the study drug the child received.

Follow-up Procedures

All enrolled children and primary caregivers will be scheduled to return to the health facility at 3 and 6 months following enrollment to collect clinical information and samples. Anthropometric measurements will be obtained from all children and caregivers at both follow up visits (height/length, weight, MUAC) and caregivers will be asked about any hospitalizations occurring since the last time the child was seen by study staff.

For peer review only

Caregivers will be provided with 400KSH (approximately $4USD) to cover the cost of their round-trip transportation.

Transportation cost will be reimbursed at each follow-up visit. If the participant does not return at their scheduled time, study staff will attempt to make contact with the primary caregiver via cell phone; if no telephone number is provided, or if the participant cannot be reached, study staff will trace the child to the household within 2 weeks of the scheduled follow-up time.

During scheduled follow-up visits, study staff will use a standardized questionnaire to ascertain history of recent illness/morbidity, post-discharge medication use including antibiotic treatment, and current condition of the child (any hospitalizations, admission and discharge date of any hospitalization, vital status, date of death if applicable). If caregivers report a hospitalization, causes of admission, medication administration, cost of admission, and length of stay will be ascertained from both caregivers and medical records, when available.

Caregivers will be encouraged, at enrollment and at each subsequent contact, to bring the child to the study health facility at any time the child is sick. Study staff will triage children to the appropriate health facility staff and will conduct a brief unscheduled visit questionnaire to ascertain adverse event information. If the unscheduled visit leads to a hospitalization, this will trigger the completion of a hospital admission form.

If at any point during follow-up a child dies, a verbal autopsy using the Population Health Metrics Research Consortium Shortened Verbal Autopsy Questionnaire.[20] If the death occurred in a hospital, data from the hospital records, including cause of death, if available, will be abstracted. If a death certificate is available, cause(s) and timing of death will be abstracted.

Final causes of re-hospitalization and death will be determined after data collection is complete by an independent adjudication committee comprised of clinicians specializing in pediatrics and infectious disease. Sources of cause of re-hospitalization (medical records and caregiver report) and causes of death (causes automatically assigned from the verbal autopsy using SmartVA-Analyze [Tariff 2.0 Method][21], hospital records, or death certificates) will be presented to the adjudication committee for final cause assignment.

Laboratory Procedures

Stool (rectal swabs), nasopharyngeal swabs and blood will be collected as described above and undergo either immediate or future laboratory testing as described in Table 3. All biological samples will be collected by staff trained in biosafety and Good Clinical Laboratory Practice (GCLP). Samples will be processed in Kenya when technology is available at the Kenya Medical Research Institute (KEMRI) Wellcome Trust or Centre for Microbiology Research [CMR]. Metagenomic analyses and/or analyses that require technology not available in Kenya will be performed at the University of Washington. If stool culture results report *Shigella* or *Salmonella* infection, the study staff will contact the child's caregiver and encourage the caregiver to bring the child back for an evaluation and potential treatment if the child is symptomatic.

Table 3. Sample storage and processing descriptions

Specimen Collected	Purpose	Tests Performed
Stool/ flocked rectal swabs	Bacterial ID and storage for AST	Fresh samples/rectal swabs will be cultured to identify *Shigella*, *Salmonella*, *Campylobacter*, and *Escherichia coli (E.coli)* using standard microbiologic methods and biochemically confirmed using bioMérieux's API® strips. All *Shigella*, *Salmonella*, and *Campylobacter* isolates, as well as a random subset of *E.coli* isolates will undergo antibiotic resistance testing using disc diffusion for the following antibiotics: amoxicillin-clavulanic acid (augmentin), ampicillin, azithromycin, chloramphenicol, ciprofloxacin, ceftriaxone, ceftazidime, cefoxitin, gentamicin, imipenem, trimethoprim-sulfamethoxazole, ceftazidime/clavulanate (ESBL), cefotaxime/clavulanate (ESBL). Categorizations of susceptible, intermediate, and resistant will be determined using zone-size cut-offs outlined in Clinical and Laboratory Standards Institute (CLSI) interpretive standards.
	Parasite detection	Fresh stool and rectal swabs will be tested for *Giardia* and *Cryptosporidium* using the immunoassay Giardia/ Cryptosporidium QUIK CHEK™.
	Storage	Stool/ flocked swabs and colonies of *E.coli*, *Shigella* spp., *Salmonella* spp., and *Campylobacter* spp. will be stored at -80°C.
Nasopharyngeal Swabs	Bacterial isolation, storage, and resistance testing	*Streptococcus pneumoniae* (*S. pneumoniae*) colonies will be isolated using standard microbiologic or molecular diagnostic protocols and susceptibility testing performed using standard microbiologic or molecular techniques. A random subset of *S. pneumoniae* isolates will undergo antimicrobial resistance testing using disc diffusion for the following antibiotics: amoxicillin-clavulanic acid (Augmentin), ampicillin, azithromycin, chloramphenicol, ciprofloxacin, ceftriaxone, ceftazidime, cefoxitin, gentamicin, imipenem, and trimethoprim-

For peer review only

		sulfamethoxazole. Categorizations of susceptible, intermediate, and resistant will be determined using zone-size cut-offs outlined in CLSI interpretive standards.
	Storage	Back-up sample and *S.pneumoniae* colonies will be will be stored at -80℃.
Blood	HIV and malaria testing	HIV testing will be performed per Kenyan National Guidelines and malaria microscopy performed using standard methods.
	Storage	Plasma and buffy coat will be stored at -80℃. Dried blood spots will be stored at room temperature.

Data Management and Confidentiality

Personal information about the participants, including medical records and data ascertained per caregiver interview, will be securely stored in files in the study offices at the study sites. Only pre-designated study staff will have access to the files. Data will be entered into an electronic database (Dacima® Electronic Data Capture) regularly by study staff. Access to the electronic database will be secured using password protected accounts for study staff. Data reports of screening, enrollment, and exclusion totals will be disseminated to the study team on a weekly basis; reports including baseline demographic characteristics, laboratory results, adherence data, and serious adverse event summaries will be distributed to study co-investigators and data monitors quarterly. Data will be regularly queried to facilitate ongoing data cleaning.

Data Analysis

Primary endpoints

The primary study endpoint is a combined outcome of mortality and hospital readmission, as re-hospitalization is highly associated with risk of subsequent poor outcome.[2] Re-hospitalizations that are a continuation of management from the previous hospitalization (such as elective blood transfusion) or that occur during enrollment procedures, due to a clinical deterioration post-discharge, will be excluded from the analysis. Loss to follow-up will be defined as non-attendance at both follow-up visits despite one month of active tracing and no clear evidence of death.

Secondary endpoints

1. *Cause-specific re-hospitalizations* assessed by questionnaire (maternal recall of diagnosis) at month 3 and month 6 follow-up visits and medical record review (discharge diagnosis). In cases when both sources are available, information from the medical record will be considered as the primary source. Separate analyses will be performed for each diagnosis: diarrhea, acute respiratory infection, malnutrition, or malaria.
2. *Mild, moderate, and severe events* that did not result in re-hospitalization, including diarrhea, vomiting, skin rash, lip swelling, difficulty breathing/wheeze, and seizure will be ascertained by caregivers identified by the study clinicians during clinical exams at scheduled follow-up visits or during unscheduled visits. Severity (grade 1-3) will be defined according to 2014 Division of AIDS (DAIDS) Table for Grading the Severity of Adult and Pediatric Adverse Events.
3. *Enteric pathogen carriage*, operationalized as presence of a bacterial pathogen-*Shigella* species (spp.), *Campylobacter* spp., or *Salmonella* spp. - or parasite- *Giardia* or *Cryptosporidium*-in stool or rectal swabs assessed at month 3 and month 6 follow-up visits.
4. *Streptococcus pneumoniae (S. pneumoniae)* isolated from nasopharyngeal swab cultures at month 3 and month 6 follow-up visits.
5. *Antibiotic resistance*, specifically resistance to azithromycin, ampicillin, augmentin, ciprofloxacin, trimethoprim-sulfamethoxazole, in *E.coli* and *S. pneumoniae* isolates, and presence of ESBL in *E.coli* isolates, from month 3 and month 6 follow-up visits.

Statistical analysis

To compare rates of re-hospitalization and mortality in the 6 months following hospital discharge among Kenyan children receiving 5-day azithromycin vs. placebo

Primary analyses will be modified intent-to-treat (mITT) based on randomization allocation to the 5-day course of azithromycin versus placebo. Cumulative incidence of death or first re-hospitalization will be compared between treatment groups using Cox proportional hazards regression. Participants will be censored at the date of their first re-hospitalization, or at the date of death. Median time to hospitalization-free survival will be

For peer review only

compared between randomization groups using Kaplan-Meier (K-M) survival analysis and associated log-rank test. If the baseline assessment of randomization reveals an imbalance in characteristics between the treatment groups, we will evaluate these variables as potential confounders in a sub-analysis secondary to the mITT. Potential baseline confounders will be added stepwise in a multivariable Cox model and maintained in the model if adjustment changes the hazard ratio by more than 10%. In per protocol analyses also secondary to the mITT, we will compare treatment effects in groups defined by self-reported adherence to the 5-day course of azithromycin (5 doses vs. < 5 doses; ≥3 doses vs. <3 doses; >1 dose vs. 1 dose only). In addition, we will conduct Cox regression and K-M survival analyses for time to mortality and time to re-hospitalization as separate endpoints to understand intervention effects on these outcomes individually. The assumption of proportional hazards will be checked in all models using graphical methods including plotting a ln(-ln(S(t))) plot for each treatment group and assessing the parallelism of the two lines and by plotting Schoenfeld residuals over time. If there is substantial missing covariate data, multiple imputation using the Markov chain Monte Carlo (MCMC) method will be used to impute covariate information. Missing outcome data (death or re-hospitalization) will not be imputed, but participants will be censored at the last follow-up visit therefore contributing some person-time to the analysis. In sensitivity analyses, we will compare treatment effects in children whose caregivers report no additional antibiotic use over follow-up and separately, who report no additional azithromycin use specifically, and in subsets of children defined by age, site, and discharge diagnosis.

To evaluate possible mechanism(s) by which azithromycin may affect morbidity and mortality, by comparing reasons for re-hospitalization and change in prevalence of pathogen carriage between the randomization arms

To evaluate the association between azithromycin and the rates of cause-specific re-hospitalizations (hospitalization due to diarrhea, acute respiratory infection, malnutrition, or malaria) we will use Anderson-Gill proportional hazards modeling with previous re-hospitalizations included as time-dependent covariates in the model to capture the dependent structure of recurrence times. Because we will not have granularity in the time points other than 3 months and 6 months for assessment of pathogen carriage, we will compare the prevalence of a bacterial and parasitic pathogens (*Shigella*, *Salmonella*, *Campylobacter, Cryptosporidium, Giardia*) at 3 and 6 months by randomization arm using generalized estimating equations (GEE) with a Poisson link, exchangeable correlation structure, and will adjust for baseline presence of a bacterial pathogen. To determine whether an observed association between the intervention and pathogen carriage wanes over time, we will test the hypothesis that the prevalence ratios comparing carriage in intervention arms are the same at the two follow-up time points using a chi-squared test.

To determine whether empiric administration of azithromycin at hospital discharge increases risk of antibiotic resistance in commensal E. coli and pneumococcal isolates from treated children and their household contacts

Among children and adult household contacts in whom commensal *E. coli* and/or *S. pneumoniae* are isolated, we will compare the proportion of isolates resistant to azithromycin, ampicillin, augmentin, ciprofloxacin, and trimethoprim-sulfamethoxazole, between randomization arms and Contact Cohorts for each arm, at 3 and 6 months using GEE with a Poisson link and exchangeable correlation structure. A chi-squared test will be used to determine whether the association between intervention arm and antibiotic resistance wanes over time. Because the likelihood of having a bacterial pathogen isolated may depend on baseline factors, including intervention arm, we will conduct secondary analyses utilizing propensity scores to account for the potential differential likelihood of having antibiotic susceptibility testing performed, which will allow us to make inference to the entire study population and their contacts. Also we will compare resistance proportions among children (as opposed to among isolates) where absence of an isolated bacteria is considered not resistant.

To identify correlates and intermediate markers of post-discharge mortality and hospital-readmission among hospitalized children

Enrollment hospital admission diagnosis, indicators of malnutrition, age, HIV-exposure and HIV-infection status, sickle cell anemia, and randomization arm will be assessed in a multivariable Cox regression model to identify correlates of the primary endpoint of death and/or hospital-readmission independent of the treatment effect. In addition, Cox regression models will also be built for correlates of mortality and correlates of re-admission individually to understand distinct cofactors for each of these outcomes.

To determine the cost-effectiveness of post-discharge administration of a 5-day course of azithromycin in settings of varying antibiotic use, re-hospitalization rates, and mortality rates

Costs analysis: We will assess the costs of all supplies, services and equipment necessary to implement the intervention (direct medical costs). The perspective will be that of the healthcare provider, i.e. Kenya's Ministry of Health. Using WHO guidelines and its ingredients approach, we will quantify the resources and associated unit costs required to deliver a 5-day course of azithromycin, organized in standard expenditure categories: personnel (salaries), supplies including drugs, equipment, services, space and overhead. We will also measure the costs of severe child hospitalizations, the costs for the different types of personnel employed (e.g. nurses/doctors) and the time demanded from them for conducting the intervention.[22] When data are missing, they will be complemented by data extracted from the literature and other available sources. Full incremental costs will be derived, with estimation of the potential healthcare cost-offset realized in avoiding severe hospitalizations. Costs will be measured in local currency (Kenyan Shilling) and converted into US$. Our main metric will be cost per child treated. Cost-effectiveness analysis (CEA): we will develop a CEA mathematical model, and estimate incremental costs and cost-effectiveness for implementation of the intervention. The model will include two components: costs (described immediately above) and health benefits. The study will provide clinical outcomes (mortality/morbidity) over a 6-month follow-up period. Subsequently, deaths averted, life-years saved and disability-adjusted life years (DALYs) averted by the intervention will be estimated. We will estimate: a) incremental costs and b) incremental cost-effectiveness of the intervention vs. status quo. *Incremental costs* are the net sum of the costs to implement the intervention compared with status quo, and the costs averted due to the decrease in severe child hospitalizations. *Incremental cost-effectiveness* ratios (ICERs) will be estimated as cost per death averted, cost per life-year saved, and cost per DALY averted. We will use recent estimates of disability weights for estimation of DALYs.[23 24] Short-term (over study follow-up i.e. 6 months) and longer-term time horizons (extrapolated to 1, 5, and 10 years) will be used. DALYs and costs will be discounted at 3% per year, consistent with CEA guidelines (undiscounted results will also be presented). Sources of uncertainty in the results will be explored in univariate and probabilistic sensitivity analysis. [25 26] Finally, we will compare our findings to CEA estimates for other health interventions in sub-Saharan Africa.[27 28]

Data and Safety Monitoring

A Data Safety and Monitoring Committee (DSMC) will be established at study initiation to monitor severe adverse events (SAEs) and to evaluate the statistical analysis plan and associated stopping rules. The DSMC includes expertise in clinical trials, statistics, child mortality assessment, ethics, and pediatric care in resource limited settings. Adverse events will be monitored by the DSMC. Monthly adverse event summaries will be sent to the DSMC safety officer and individual-child SAE forms, which include detailed medication history to evaluate possible drug interactions, will be sent to the safety officer per request. Each SAE will be assigned the plausibility of relatedness to study drug by study PIs. The data will not be presented by intervention group unless requested by DSMB safety officer. These reports will be descriptive (no statistical analyses). The DSMC will make recommendations regarding any imbalances in safety outcomes.

A single interim analysis for re-hospitalization-free survival will be prepared by the study statistician using O'Brien-Fleming boundaries for benefit and harm when 50% of expected person time (350 child-years) has been accrued. Assuming 157 events will be available at half of the person-time accrual, a z-score critical value of 2.797, or *p*-value < 0.005, from a Kaplan Meier log-rank test will determine the cut-off of statistical significance. A symmetric boundary will be used for benefit and harm. The DSMC will review this analysis and make a determination about study continuation. Futility will not be a basis for stopping rules because of the trials' value in understanding mechanisms of post-discharge worsening and antibiotic resistance. Assuming the DSMC decides to continue the trial after the interim analysis, an alpha of 0.045 will be used as the statistical significance boundary at the final analysis.

Statistical Power

To compare rates of re-hospitalization and mortality in the 6 months following hospital discharge among Kenyan children receiving 5-day azithromycin vs. placebo

The total sample size required was calculated for the primary endpoint of time to death or hospital re-admission within the 6 month post-discharge period, assuming an alpha level of 0.05, power of 0.80, and a

ratio of treatment to placebo random assignment of 1:1. In SSA, it is estimated that 2-15% of children aged less than 5 years died within 6 months of hospital discharge and 15.5% of children who survived discharge from the district hospital were re-admitted with the same diagnosis within 6-months.[2 4 9] Assuming that an additional 5-10% of children are re-admitted for other conditions, we expect that re-hospitalizations will occur in 20.5 to 30.5% of children enrolled in the study. Combined with our expected fatality rate (2-15%), we expect the cumulative incidence of the combined endpoint to range from 22.5 to 45.5%.[9] Based on a previous trial of mass drug administration of a single dose of azithromycin in which a single dose of the antibiotic was associated with a 49% reduction in risk of death, we calculated sample sizes using estimates of reduction in risk ranging from 30-50% with the cumulative incidence range of 22.5 to 45.5% in the placebo-treated group, and found the sample size required ranged from 90 to 550 children per treatment arm.[16] Using the most conservative estimates of a hazard ratio of 0.70 and 22.5% prevalence of re-admission/death, we need to enroll 1100 children in the study (550 per arm) to achieve adequate power. We will recruit an additional 300 children (≈20%) to account for possible loss to follow-up, resulting in a total planned enrollment of 1400 children, or 700 per treatment group. When considering mortality alone, and estimated mortality ranges of 2-17% among place-treated children, we will have >80% power to detect hazard ratios ≤0.5 for mortality rates of ≥ 8% and hazard ratios ≤0.6 for mortality rates ≥11% (Figure 1).

To evaluate possible mechanism(s) by which azithromycin may affect morbidity and mortality, by comparing reasons for re-hospitalization and prevalence of pathogen carriage between the randomization arms

We calculated the minimum detectable association between treatment arm and cause-specific re-hospitalizations (hospitalization due to diarrhea, acute respiratory infection, malnutrition, or malaria vs. any other) among enrolled children, assuming an alpha level of 0.05, power of 0.80, and a ratio of treatment to placebo of 1:1. Based on data from Kenya, re-hospitalization rates due to specific causes ranged from approximately 0.5% to 5.7% in the 6 month post-discharge period.[4] By not conditioning on the child having the same diagnosis as the initial hospitalization, we expect the cumulative incidence of cause-specific re-hospitalizations to range from 2.5% to 10%. With this range of outcome rates, we will be able to detect hazard ratios of 0.48 to 0.70 for the effect of azithromycin on specific severe morbidities.

We expect 56% of children in the placebo group to have *S. pneumoniae* isolated from nasopharyngeal swabs, providing ≥80% power to detect a prevalence ratio of 0.85 (or 1.15) between the two treatment arms at each time point.[29-31] Based on prevalences of *Shigella*, *Salmonella*, *Campylobacter, Cryptosporidium, Giardia* among asymptomatic children in Western Kenya, we expect 10% of children in the placebo group to have a bacterial pathogen isolated at each time point, resulting in ≥80% power to detect differences in enteric pathogen prevalences of 0.67 (1.49) at each time point.[32]

To determine whether empiric administration of azithromycin at hospital discharge increases risk of antibiotic resistance in commensal E. coli and S. pneumoniae isolates from treated children and their household contacts

We will select a random selection of 400 *E. coli* and 400 *S. pneumoniae* isolates (200 per arm) for β-lactam and macrolide resistance testing at each timepoint. We will also store all *S. pneumoniae*, *E. coli* isolates and other isolated bacteria from stool for potential future testing in the event that resistance prevalence is lower than expected. As shown in Table 4, we will have > 80% power to detect prevalence ratios > 1.1, with an ability to detect the smallest effect sizes when the prevalence of resistance in the placebo group is highest. We will enroll 300 adults in the Contact Cohort for *E. coli* and *S. pneumoniae* isolation. We expect *E.coli* to be isolated from all adults and *S.pnuemoniae* isolated from between 5-55%.[29 33 34] Assuming an alpha of .05, a 1:1 ratio of testable isolates, and a prevalence of resistance of 50% in the placebo arm, we will have 80% power to detect a 1.4-fold higher prevalence to 1.9-fold higher resistance prevalence in the contacts of azithromycin-treated children.

Table 4. Power (%) to detect prevalence ratios of macrolide and β-lactamase resistance in 200 *E.coli* and 200 *S.pneumoniae* isolates per treatment group

		**Resistance prevalence (%) in placebo group**						
		**10**	**20**	**30**	**40**	**50**	**60**	**70**
**Resistance prevalence (%) in azithromycin group**	**10**							
	**20**	80						
	**30**	>99	64					
	**40**	>99	99	55				
	**50**	>99	>99	98	48			
	**60**	>99	>99	>99	>99	52		
	**70**	>99	>99	>99	>99	98	55	
	**80**	>99	>99	>99	>99	>99	99	64

To identify correlates and intermediate markers of post-discharge mortality and hospital-readmission among hospitalized Kenyan children

Conservatively estimating a 20% loss-to-follow-up rate in the RCT and a cumulative incidence of death or re-hospitalization of 22.5%, we will have >80% power to detect hazard ratios ≥1.3 between correlates and the outcome with exposure prevalences of ≥20% or more and hazard ratios ≥1.5 for exposure prevalences <20%.

Study timeline

The trial began on June 28, 2016 and participant recruitment and follow-up will continue over a 36-month period, with anticipated final follow-up visit(s) occurring in June 2019. Primary analyses will be complete by February 2020.

Potential Challenges and Limitations

In order to ensure adequate power to detect a discernable clinically relevant difference between study groups in the primary outcome, we have combined hospital readmission with death. Preliminary studies suggest that sufficient numbers of children will reach this combined outcome. However, we have incorporated an interim analysis by the DSMB to review the accrued data and an adapted sample size could be considered if the combined event frequency is less than predicted. It is possible that since most children receive antibiotics during hospitalization, the benefit anticipated with the use of azithromycin based on previous trials of mass drug administration will not be observed. However, most hospitalized children are treated with penicillins, cephalosporins, gentamicin, or cotrimoxazole while in hospital and the broad spectrum of activity (including malaria prevention) and long half-life of azithromycin suggest that there may be additive treatment and/or prophylactic benefit. Similarly, children may receive azithromycin during follow up - either as treatment for an illness or because the caregiver sought out azithromycin upon learning of the hypothesis – and this azithromycin use may lead to contamination in the placebo-arm. After discharge, it is difficult to ensure adherence with the full 5-day treatment course. We will measure adherence using three different measures (text message responses, bottle check boxes, and caregiver-report at follow-up visits) although all are limited by caregiver-report. In addition, the mortality benefit of azithromycin observed in Ethiopia was from a single dose and in this study the first dose will be directly observed.[16] While relying on caregiver report of mortality and morbidity may lead to bias due to outcome misclassification, this misclassification should not differ between randomization arms and therefore will be non-differential. Further hospital records will be used when available to determine diagnoses. Finally, resistance prevalence may be lower than predicted, limiting power to detect clinically relevant differences in resistance prevalence between the intervention arms. We will store all isolates in the event that a greater number of isolates are needed for antibiotic resistance testing.

Ethics and Dissemination

This study has received IRB approval by the University of Washington Human Subjects Division (HSD), KEMRI Scientific and Ethics Review Unit (SERU), and the Kenya Pharmacy and Poisons Board. The clinical trial is also registered with clinicaltrials.gov (NCT02414399). Any modifications to the study protocol or consent materials will be submitted for approval all regulatory authorities before implementation. The study is being externally monitored and a data safety and monitoring committee has been assembled to monitor patient safety and to evaluate the efficacy of the intervention. Results of this study will be disseminated by publication in a peer-reviewed scientific journal, presented at relevant academic conferences, and amongst participating partners and health facilities in Kenya.

Author's contributions

JLW, PBP, GJS, BAR, BOS, and RN conceived of this trial and developed of this study protocol. JLW and BOS are study co-Principal Investigators and PBP is the Project Director; BAR oversaw the statistical analyses plans; JBB developed the CEA plan; KDT developed procedures for ascertaining and reporting SAEs; CJM developed procedures related to blood specimen procedures and drug adherence measurement. GJS, CJM, RN, and PBP provide scientific expertise. RLB and MA are involved in collection and management of the data. MA and PBP coordinate and oversee implementation of all clinical study procedures and SK, with assistance

For peer review only

from DR, oversees all laboratory procedures. All authors contributed to the development of this manuscript and/or study procedures, and to reading and approving the final version for publication.

Funding statement
This work was funded by the National Institutes of Health Eunice Kennedy Shriver National Institute of Child Health & Human Development, grant number R01 HD079695.

Acknowledgements
Pfizer donated the Zithromax® to be used in this clinical trial, and Copan Diagnostics donated all rectal swabs and Cary-Blair media. Investigators from KEMRI-Wellcome Trust Kilifi, Jay Berkley, Anthony Scott, Joseph Waichungo, Angela Karani, Donald Akech, and Horace Gumba provided microbiology expertise and training in nasopharyngeal swab collection, STGG media preparation, and laboratory quality assurance and control. Liru Meshack Wekesa and George Bogonko provide clinical expertise and facilitate the study's integration into pediatric wards at two of the study hospitals, Homa Bay District and Kisii Teaching & Referral Hospital, respectively. Alex Awuor and Caleb Okonji, with the support of Richard Omore, provided training in anthropometric measurement. We are extremely thankful to Dr. Philip Walson who developed azithromycin dosing regimens. Hannah Atlas and Stephanie Belanger contributed to the standard operating procedure and case report form development and implementation. Gillian Levine played an invaluable role in the proposal development.

Competing interests statement
None of the authors or study co-investigators have any competing interests to declare.

Figure Legend
Figure 1. Power and detectable hazard ratios assuming a range of mortality rates from 2-17%

REFERENCES

1. Collaborators GBDCM. Global, regional, national, and selected subnational levels of stillbirths, neonatal, infant, and under-5 mortality, 1980-2015: a systematic analysis for the Global Burden of Disease Study 2015. *Lancet* 2016;388(10053):1725-74. doi: 10.1016/S0140-6736(16)31575-6
2. Moisi JC, Gatakaa H, Berkley JA, et al. Excess child mortality after discharge from hospital in Kilifi, Kenya: a retrospective cohort analysis. *Bull World Health Organ* 2011;89(10):725-32, 32A. doi: 10.2471/BLT.11.089235
3. Veirum JE, Sodeman M, Biai S, et al. Increased mortality in the year following discharge from a paediatric ward in Bissau, Guinea-Bissau. *Acta Paediatr* 2007;96(12):1832-8. doi: 10.1111/j.1651-2227.2007.00562.x [published Online First: 2007/11/16]
4. Snow RW, Howard SC, Mung'Ala-Odera V, et al. Paediatric survival and re-admission risks following hospitalization on the Kenyan coast. *Trop Med Int Health* 2000;5(5):377-83. [published Online First: 2000/07/08]
5. Zucker JR, Lackritz EM, Ruebush TK, 2nd, et al. Childhood mortality during and after hospitalization in western Kenya: effect of malaria treatment regimens. *Am J Trop Med Hyg* 1996;55(6):655-60. [published Online First: 1996/12/01]
6. Islam MA, Rahman MM, Mahalanabis D, et al. Death in a diarrhoeal cohort of infants and young children soon after discharge from hospital: risk factors and causes by verbal autopsy. *J Trop Pediatr* 1996;42(6):342-7. [published Online First: 1996/12/01]
7. Villamor E, Misegades L, Fataki MR, et al. Child mortality in relation to HIV infection, nutritional status, and socio-economic background. *Int J Epidemiol* 2005;34(1):61-8. doi: 10.1093/ije/dyh378 [published Online First: 2005/01/15]
8. Phiri KS, Calis JC, Faragher B, et al. Long term outcome of severe anaemia in Malawian children. *PLoS One* 2008;3(8):e2903. doi: 10.1371/journal.pone.0002903 [published Online First: 2008/08/07]

For peer review only

9. Wiens MO, Pawluk S, Kissoon N, et al. Pediatric post-discharge mortality in resource poor countries: a systematic review. *PLoS One* 2013;8(6):e66698. doi: 10.1371/journal.pone.0066698 [published Online First: 2013/07/05]
10. Bwakura-Dangarembizi M, Kendall L, Bakeera-Kitaka S, et al. A randomized trial of prolonged co-trimoxazole in HIV-infected children in Africa. *The New England journal of medicine* 2014;370(1):41-53. doi: 10.1056/NEJMoa1214901
11. Walker AS, Mulenga V, Ford D, et al. The impact of daily cotrimoxazole prophylaxis and antiretroviral therapy on mortality and hospital admissions in HIV-infected Zambian children. *Clin Infect Dis* 2007;44(10):1361-7. doi: 10.1086/515396 [published Online First: 2007/04/20]
12. Chintu C, Bhat GJ, Walker AS, et al. Co-trimoxazole as prophylaxis against opportunistic infections in HIV-infected Zambian children (CHAP): a double-blind randomised placebo-controlled trial. *Lancet* 2004;364(9448):1865-71. doi: 10.1016/S0140-6736(04)17442-4 [published Online First: 2004/11/24]
13. Trehan I, Goldbach HS, LaGrone LN, et al. Antibiotics as part of the management of severe acute malnutrition. *The New England journal of medicine* 2013;368(5):425-35. doi: 10.1056/NEJMoa1202851
14. Lockman S, Hughes M, Powis K, et al. Effect of co-trimoxazole on mortality in HIV-exposed but uninfected children in Botswana (the Mpepu Study): a double-blind, randomised, placebo-controlled trial. *Lancet Glob Health* 2017;5(5):e491-e500. doi: 10.1016/s2214-109x(17)30143-2 [published Online First: 2017/04/12]
15. Berkley JA, Ngari M, Thitiri J, et al. Daily co-trimoxazole prophylaxis to prevent mortality in children with complicated severe acute malnutrition: a multicentre, double-blind, randomised placebo-controlled trial. *Lancet Glob Health* 2016;4(7):e464-73. doi: 10.1016/S2214-109X(16)30096-1
16. Porco TC, Gebre T, Ayele B, et al. Effect of mass distribution of azithromycin for trachoma control on overall mortality in Ethiopian children: a randomized trial. *JAMA* 2009;302(9):962-8. doi: 10.1001/jama.2009.1266 [published Online First: 2009/09/03]
17. Keenan JD, Ayele B, Gebre T, et al. Childhood mortality in a cohort treated with mass azithromycin for trachoma. *Clin Infect Dis* 2011;52(7):883-8. doi: 10.1093/cid/cir069 [published Online First: 2011/03/24]
18. O'Brien KL, Nohynek H, World Health Organization Pneumococcal Vaccine Trials Carraige Working G. Report from a WHO working group: standard method for detecting upper respiratory carriage of Streptococcus pneumoniae. *Pediatr Infect Dis J* 2003;22(2):133-40. doi: 10.1097/01.inf.0000048676.93549.d1
19. Satzke C, Turner P, Virolainen-Julkunen A, et al. Standard method for detecting upper respiratory carriage of Streptococcus pneumoniae: updated recommendations from the World Health Organization Pneumococcal Carriage Working Group. *Vaccine* 2013;32(1):165-79. doi: 10.1016/j.vaccine.2013.08.062
20. Serina P, Riley I, Stewart A, et al. A shortened verbal autopsy instrument for use in routine mortality surveillance systems. *BMC Med* 2015;13:302. doi: 10.1186/s12916-015-0528-8
21. Serina P, Riley I, Stewart A, et al. Improving performance of the Tariff Method for assigning causes of death to verbal autopsies. *BMC Med* 2015;13:291. doi: 10.1186/s12916-015-0527-9
22. WHO. Making choices in health: WHO guide to cost-effectiveness analysis. 2003.
23. Murray CJ, Vos T, Lozano R, et al. Disability-adjusted life years (DALYs) for 291 diseases and injuries in 21 regions, 1990-2010: a systematic analysis for the Global Burden of Disease Study 2010. *Lancet* 2012;380(9859):2197-223. doi: 10.1016/s0140-6736(12)61689-4 [published Online First: 2012/12/19]
24. Salomon JA, Vos T, Hogan DR, et al. Common values in assessing health outcomes from disease and injury: disability weights measurement study for the Global Burden of Disease Study 2010. *Lancet* 2012;380(9859):2129-43. doi: 10.1016/s0140-6736(12)61680-8 [published Online First: 2012/12/19]
25. Gold M, Siegel JE, Russell LB, Weinstein MC. Cost-effectiveness in health and medicine. Oxford University Press: New York 1996.
26. Drummond MF, Torrance GW, O'Brien BJ, Stoddart GL. Methods for the economic evaluation of healthcare programmes, 3rd edition. Oxford University Press: New York 2005.
27. Laxminarayan R, Mills AJ, Breman JG, et al. Advancement of global health: key messages from the Disease Control Priorities Project. *Lancet* 2006;367(9517):1193-208. doi: 10.1016/s0140-6736(06)68440-7 [published Online First: 2006/04/18]
28. Jamison DT, Measham A, Alleyne G, Claeson M, et al. Disease Control Priorities in Developing Countries, 2nd edition. Oxford University Press, 2006.

For peer review only

29. Abdullahi O, Karani A, Tigoi CC, et al. The prevalence and risk factors for pneumococcal colonization of the nasopharynx among children in Kilifi District, Kenya. *PLoS One* 2012;7(2):e30787. doi: 10.1371/journal.pone.0030787 [published Online First: 2012/03/01]
30. Rodrigues F, Foster D, Caramelo F, et al. Progressive changes in pneumococcal carriage in children attending daycare in Portugal after 6 years of gradual conjugate vaccine introduction show falls in most residual vaccine serotypes but no net replacement or trends in diversity. *Vaccine* 2012;30(26):3951-6. doi: 10.1016/j.vaccine.2012.03.058 [published Online First: 2012/04/07]
31. Grivea IN, Tsantouli AG, Michoula AN, et al. Dynamics of Streptococcus pneumoniae nasopharyngeal carriage with high heptavalent pneumococcal conjugate vaccine coverage in Central Greece. *Vaccine* 2011;29(48):8882-7. doi: 10.1016/j.vaccine.2011.09.074 [published Online First: 2011/10/04]
32. Skalet AH, Cevallos V, Ayele B, et al. Antibiotic selection pressure and macrolide resistance in nasopharyngeal Streptococcus pneumoniae: a cluster-randomized clinical trial. *PLoS Med* 2010;7(12):e1000377. doi: 10.1371/journal.pmed.1000377 [published Online First: 2010/12/24]
33. Adetifa IM, Antonio M, Okoromah CA, et al. Pre-vaccination nasopharyngeal pneumococcal carriage in a Nigerian population: epidemiology and population biology. *PLoS One* 2012;7(1):e30548. doi: 10.1371/journal.pone.0030548 [published Online First: 2012/02/01]
34. Hill PC, Akisanya A, Sankareh K, et al. Nasopharyngeal carriage of Streptococcus pneumoniae in Gambian villagers. *Clin Infect Dis* 2006;43(6):673-9. doi: 10.1086/506941 [published Online First: 2006/08/17]

For peer review only

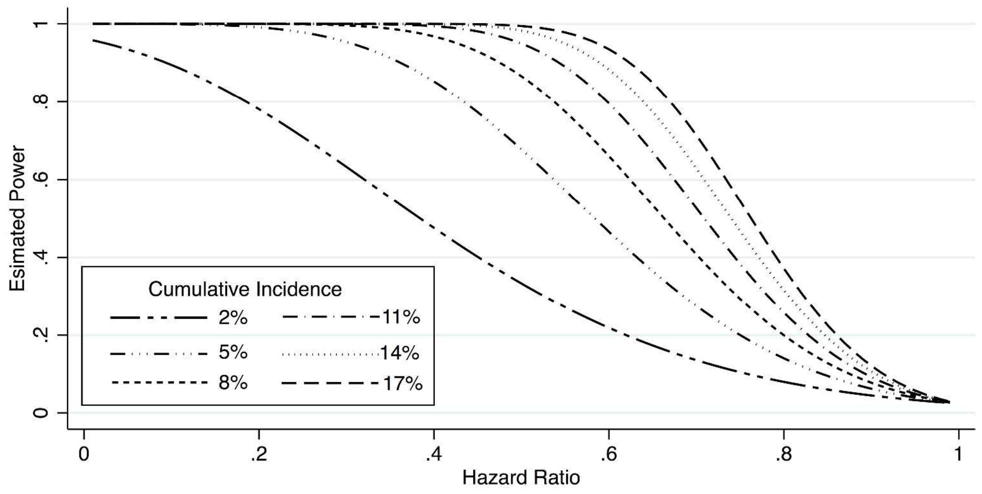

Figure 1. Power and detectable hazard ratios assuming a range of mortality rates from 2-17%

644x332mm (300 x 300 DPI)

STANDARD PROTOCOL ITEMS: RECOMMENDATIONS FOR INTERVENTIONAL TRIALS

SPIRIT 2013 Checklist: Recommended items to address in a clinical trial protocol and related documents*

Section/item	Item No	Description	Addressed on page number
**Administrative information**			
Title	1	Descriptive title identifying the study design, population, interventions, and, if applicable, trial acronym	______________
Trial registration	2a	Trial identifier and registry name. If not yet registered, name of intended registry	______________
	2b	All items from the World Health Organization Trial Registration Data Set	______________
Protocol version	3	Date and version identifier	______________
Funding	4	Sources and types of financial, material, and other support	______________
Roles and responsibilities	5a	Names, affiliations, and roles of protocol contributors	______________
	5b	Name and contact information for the trial sponsor	______________
	5c	Role of study sponsor and funders, if any, in study design; collection, management, analysis, and interpretation of data; writing of the report; and the decision to submit the report for publication, including whether they will have ultimate authority over any of these activities	______________
	5d	Composition, roles, and responsibilities of the coordinating centre, steering committee, endpoint adjudication committee, data management team, and other individuals or groups overseeing the trial, if applicable (see Item 21a for data monitoring committee)	______________

For peer review only

Introduction

Background and rationale	6a	Description of research question and justification for undertaking the trial, including summary of relevant studies (published and unpublished) examining benefits and harms for each intervention	______________
	6b	Explanation for choice of comparators	______________
Objectives	7	Specific objectives or hypotheses	______________
Trial design	8	Description of trial design including type of trial (eg, parallel group, crossover, factorial, single group), allocation ratio, and framework (eg, superiority, equivalence, noninferiority, exploratory)	______________

Methods: Participants, interventions, and outcomes

Study setting	9	Description of study settings (eg, community clinic, academic hospital) and list of countries where data will be collected. Reference to where list of study sites can be obtained	______________
Eligibility criteria	10	Inclusion and exclusion criteria for participants. If applicable, eligibility criteria for study centres and individuals who will perform the interventions (eg, surgeons, psychotherapists)	______________
Interventions	11a	Interventions for each group with sufficient detail to allow replication, including how and when they will be administered	______________
	11b	Criteria for discontinuing or modifying allocated interventions for a given trial participant (eg, drug dose change in response to harms, participant request, or improving/worsening disease)	______________
	11c	Strategies to improve adherence to intervention protocols, and any procedures for monitoring adherence (eg, drug tablet return, laboratory tests)	______________
	11d	Relevant concomitant care and interventions that are permitted or prohibited during the trial	______________
Outcomes	12	Primary, secondary, and other outcomes, including the specific measurement variable (eg, systolic blood pressure), analysis metric (eg, change from baseline, final value, time to event), method of aggregation (eg, median, proportion), and time point for each outcome. Explanation of the clinical relevance of chosen efficacy and harm outcomes is strongly recommended	______________
Participant timeline	13	Time schedule of enrolment, interventions (including any run-ins and washouts), assessments, and visits for participants. A schematic diagram is highly recommended (see Figure)	______________

For peer review only - http://bmjopen.bmj.com/site/about/guidelines.xhtml

Sample size	14	Estimated number of participants needed to achieve study objectives and how it was determined, including clinical and statistical assumptions supporting any sample size calculations	______________
Recruitment	15	Strategies for achieving adequate participant enrolment to reach target sample size	______________

Methods: Assignment of interventions (for controlled trials)

Allocation:			
Sequence generation	16a	Method of generating the allocation sequence (eg, computer-generated random numbers), and list of any factors for stratification. To reduce predictability of a random sequence, details of any planned restriction (eg, blocking) should be provided in a separate document that is unavailable to those who enrol participants or assign interventions	______________
Allocation concealment mechanism	16b	Mechanism of implementing the allocation sequence (eg, central telephone; sequentially numbered, opaque, sealed envelopes), describing any steps to conceal the sequence until interventions are assigned	______________
Implementation	16c	Who will generate the allocation sequence, who will enrol participants, and who will assign participants to interventions	______________
Blinding (masking)	17a	Who will be blinded after assignment to interventions (eg, trial participants, care providers, outcome assessors, data analysts), and how	______________
	17b	If blinded, circumstances under which unblinding is permissible, and procedure for revealing a participant's allocated intervention during the trial	______________

Methods: Data collection, management, and analysis

Data collection methods	18a	Plans for assessment and collection of outcome, baseline, and other trial data, including any related processes to promote data quality (eg, duplicate measurements, training of assessors) and a description of study instruments (eg, questionnaires, laboratory tests) along with their reliability and validity, if known. Reference to where data collection forms can be found, if not in the protocol	______________
	18b	Plans to promote participant retention and complete follow-up, including list of any outcome data to be collected for participants who discontinue or deviate from intervention protocols	______________

Data management	19	Plans for data entry, coding, security, and storage, including any related processes to promote data quality (eg, double data entry; range checks for data values). Reference to where details of data management procedures can be found, if not in the protocol	______________
Statistical methods	20a	Statistical methods for analysing primary and secondary outcomes. Reference to where other details of the statistical analysis plan can be found, if not in the protocol	______________
	20b	Methods for any additional analyses (eg, subgroup and adjusted analyses)	______________
	20c	Definition of analysis population relating to protocol non-adherence (eg, as randomised analysis), and any statistical methods to handle missing data (eg, multiple imputation)	______________
**Methods: Monitoring**			
Data monitoring	21a	Composition of data monitoring committee (DMC); summary of its role and reporting structure; statement of whether it is independent from the sponsor and competing interests; and reference to where further details about its charter can be found, if not in the protocol. Alternatively, an explanation of why a DMC is not needed	______________
	21b	Description of any interim analyses and stopping guidelines, including who will have access to these interim results and make the final decision to terminate the trial	______________
Harms	22	Plans for collecting, assessing, reporting, and managing solicited and spontaneously reported adverse events and other unintended effects of trial interventions or trial conduct	______________
Auditing	23	Frequency and procedures for auditing trial conduct, if any, and whether the process will be independent from investigators and the sponsor	______________
**Ethics and dissemination**			
Research ethics approval	24	Plans for seeking research ethics committee/institutional review board (REC/IRB) approval	______________
Protocol amendments	25	Plans for communicating important protocol modifications (eg, changes to eligibility criteria, outcomes, analyses) to relevant parties (eg, investigators, REC/IRBs, trial participants, trial registries, journals, regulators)	______________

For peer review only - http://bmjopen.bmj.com/site/about/guidelines.xhtml

Consent or assent	26a	Who will obtain informed consent or assent from potential trial participants or authorised surrogates, and how (see Item 32)	______________
	26b	Additional consent provisions for collection and use of participant data and biological specimens in ancillary studies, if applicable	______________
Confidentiality	27	How personal information about potential and enrolled participants will be collected, shared, and maintained in order to protect confidentiality before, during, and after the trial	______________
Declaration of interests	28	Financial and other competing interests for principal investigators for the overall trial and each study site	______________
Access to data	29	Statement of who will have access to the final trial dataset, and disclosure of contractual agreements that limit such access for investigators	______________
Ancillary and post-trial care	30	Provisions, if any, for ancillary and post-trial care, and for compensation to those who suffer harm from trial participation	______________
Dissemination policy	31a	Plans for investigators and sponsor to communicate trial results to participants, healthcare professionals, the public, and other relevant groups (eg, via publication, reporting in results databases, or other data sharing arrangements), including any publication restrictions	______________
	31b	Authorship eligibility guidelines and any intended use of professional writers	______________
	31c	Plans, if any, for granting public access to the full protocol, participant-level dataset, and statistical code	______________
**Appendices**			
Informed consent materials	32	Model consent form and other related documentation given to participants and authorised surrogates	______________
Biological specimens	33	Plans for collection, laboratory evaluation, and storage of biological specimens for genetic or molecular analysis in the current trial and for future use in ancillary studies, if applicable	______________

*It is strongly recommended that this checklist be read in conjunction with the SPIRIT 2013 Explanation & Elaboration for important clarification on the items. Amendments to the protocol should be tracked and dated. The SPIRIT checklist is copyrighted by the SPIRIT Group under the Creative Commons "Attribution-NonCommercial-NoDerivs 3.0 Unported" license.

For peer review only - http://bmjopen.bmj.com/site/about/guidelines.xhtml
